# Early detection of myocardial changes with and without dexrazoxane using serial magnetic resonance imaging in a pre-clinical mouse model

**DOI:** 10.1186/s40959-021-00109-8

**Published:** 2021-06-16

**Authors:** Cory V. Noel, Nino Rainusso, Matthew Robertson, Jonathan Romero, Prakash Masand, Cristian Coarfa, Robia Pautler

**Affiliations:** 1 Pediatric Cardiology of Alaska, Seattle Children’s Hospital - Division of Pediatric Cardiology, Anchorage, AK USA; 2 Division of Pediatric Hematology and Oncology, Baylor College of Medicine, Houston, USA; 3 Dan L Duncan Comprehensive Cancer Center, Baylor College of Medicine, Houston, USA; 4 Baylor College of Medicine Small Animal Imaging Facility, Houston, USA; 5 Texas Children’s Hospital Pediatric Radiology, Houston, USA; 6 Department of Molecular and Cellular Biology, Baylor College of Medicine, Houston, USA

**Keywords:** Cardiac MRI, Anthracycline, Dexrazoxane, Pre-clinical, Early detection, Cardiotoxicity

## Abstract

**Background:**

Cancer therapy-related cardiac dysfunction may occur in pediatric cancer survivors. Identification of early markers of myocardial damage secondary to anthracycline exposure is crucial to develop strategies that may ameliorate this complication.

**Objectives:**

The purpose of this study was to identify early myocardial changes induced by doxorubicin with and without cardioprotection using dexrazoxane detected by serial cardiac magnetic resonance imaging (CMR) in a pre-clinical mouse model.

**Methods:**

Serial CMR examinations were performed in 90 mice distributed in 3 groups: 45 received doxorubicin (DOX group), 30 mice received doxorubicin with dexrazoxane (DOX/DEX group) and 15 mice received saline injections (control group). We obtained the following CMR parameters in all mice: T2, extracellular volume quantification (ECV), myocardial deformation, and functional quantification.

**Results:**

Myocardial edema assessed by T2 time was the earliest parameter demonstrating evidence of myocardial injury, most notable in the DOX group at week 4 and 8 compared with DOX/DEX group. Similarly, global longitudinal strain was abnormal in both the DOX and DOX/DEX groups. However, this change persisted only in the DOX group. The ECV was significantly elevated in the DOX group at the final CMR, while only minimally elevated in the DOX/DEX group. The right and left ejection fraction was decreased, along with the mass to volume ratio in the DOX group. The T2 time, ECV, and deformation correlated with ejection fraction and left ventricular volume.

**Conclusions:**

T2 time and deformation by CMR identifies early myocardial injury from anthracyclines. Dexrazoxne did not prevent the initial edema, but the inflammatory changes were not sustained. CMR may be useful for early detection of cardiac dysfunction. Serial CMR demonstrates dexrazoxane minimizes cardiac dysfunction and aids recovery in a mouse model.

**Supplementary Information:**

The online version contains supplementary material available at 10.1186/s40959-021-00109-8.

## Translational perspective

Cancer therapy-related cardiac dysfunction is one of the leading causes of morbidity and mortality in pediatric cancer survivors. The identification of early changes in the myocardium secondary to cancer therapy may assist with the development of different strategies to mitigate this effect. Our study utilizing a pre-clinical mouse model demonstrated that early myocardial changes occur with doxorubicin even when it is administrated in conjunction with dexrazoxane. However, chronic inflammation and subsequent ventricular dysfunction due to doxorubicin may be mitigate by cardioprotection with dexrazoxane. In addition, our study included GLS in a pre-clinical mouse model as one of the parameters to evaluate myocardial damage after anthracycline exposure. We observed that GLS was impaired in mice that received doxorubicin as it has been described in cancer patients. Our results support previous studies that advocate for a widespread use of cardiac MRI in pediatric cancer patients in larger clinical to monitor for the deleterious cardiotoxic effects exert by chemotherapy.

## Introduction

The rates of survival of pediatric cancers have risen considerably over the past four decades. One reason for improvement is the use of combination chemotherapy. Several anthracyclines, a class of chemotherapy drugs, have become the mainstay of treatment for different pediatric cancers [1]. However, the use of anthracyclines is limited by their known deleterious cardiovascular effects [2]. The most common effect of cancer therapy-related cardiac dysfunction (CTRCD) is left ventricular systolic dysfunction as measured by decreased ejection fraction (EF) [3]. The significance of CTRCD cannot be understated, as the leading cause of morbidity and mortality in childhood cancer survivors excluding cancer recurrence is cardiovascular disease [4]. Once CTRCD is diagnosed in the form of heart failure, it has a predicted two-year survival of just 40% [5]. The cardiotoxic effects of anthracyclines, such as doxorubicin or mitoxantrone, can be mitigated with the use of dexrazoxane before the administration of anthracyclines. Dexrazoxane binds topoisomerase IIβ to increase its degradation and prevent cardiomyocyte apoptosis. In addition, dexrazoxane decreases the formation of reactive oxygen species in the myocardium which also have deleterious effects on cardiomyocytes [6, 7]. Although there is reluctance to administer dexrazoxane to pediatric patients, a recent study from the Children’s Oncology Group demonstrated improvement in reducing the left ventricular systolic dysfunction and a suggestion of reducing treatment-related mortality [8].

Trans-thoracic echocardiography (TTE) remains the primary imaging modality to monitor for signs of CTRCD due to its wide availability, lack or radiation exposure, capacity to assess hemodynamics and lower cost. However, TTE is unable to detect myocardial edema and fibrosis resulting from cardiomyocyte injury, and typically evaluates for CTRCD by measures of global systolic function such as left ventricle shortening fraction (LVSF) or left ventricle ejection fraction (LVEF). In addition, evaluation of cardiotoxicity with TTE has a predilection to overestimate the LVEF, and may fail to identify patients with early left ventricle dysfunction [9, 10]. Therefore, other imaging modalities, such as nuclear cardiac imaging or cardiac magnetic resonance (CMR), have been proposed for earlier detection of cardiotoxicity in patients exposed to anthracyclines [11].

Cardiac magnetic resonance imaging has superior quantification of left ventricle (LV) size and LVEF compared to traditional 2-dimensional TTE, and indicates the degree of myocardial edema and extracellular volume (ECV) fraction secondary to myocardial damage. Therefore, CMR could be considered the imaging reference standard to evaluate for heart failure and detect early stages of this disease [12–14]. A serial CMR characterization of doxorubicin-induced cardiotoxicity from pre-treatment to left ventricle dysfunction and heart failure in an animal model (pig) has been recently described [15]. However, the cardioprotective effect and the CMR imaging changes exerted by dexrazoxane have not been completely elucidated. Here, we used a pre-clinical mouse model of doxorubicin-induced cardiotoxicity to determine the predictive value of the earliest CMR markers of myocardial damage (myocardial edema, fibrosis and strain) and the changes associated with dexrazoxane administration.

## Methods

This study was approved by Baylor College of Medicine Institutional Animal Care Use Committee. This was a longitudinal study of 90 wild-type C57BL/6 mice (Jackson Laboratories, Bar Harbor, ME) aged 6–8 weeks randomized to doxorubicin (DOX group, *n* = 45), doxorubicin + dexrazoxane (DOX/DEX group, *n* = 30) or placebo with saline injection (control group, *n* = 15). The DOX group received doxorubicin 3 mg/kg once per week by intraperitoneal injection for a total of 6 weeks. The DOX/DEX group received doxorubicin 3 mg/kg plus dexrazoxane 30 mg/kg intraperitoneal injection once per week for a total of 6 weeks. These doses were chosen to simulate the development of anthracycline-induced cardiotoxicity. The control group received intraperitoneal saline (same volume as the DOX group) once per week for 6 weeks.

The key measures assessed by the CMR were LV and right ventricle (RV) size, LVEF, RVEF, myocardial edema evaluated by average T2-relaxation time, myocardial fibrosis assessed by ECV and myocardial strain. The CMR variables were assessed at 4 weeks (during chemotherapy administration), 8 weeks (2 weeks after conclusion of chemotherapy) and 12 weeks (6 weeks after conclusion of chemotherapy). Images were obtained 48–72 h after dexrazoxane and doxorubicin administration on week 4. The primary outcome of the study was the presence of ventricular dysfunction, which was defined as decrease in LVEF or RVEF below 50%. The CMR parameters were analyzed by individuals blinded to the treatment groups.

### Cardiac magnetic resonance imaging

Serial CMR examinations were performed on a Bruker 9.4-T CMR imaging machine as previously described [16, 17] For the CMR examinations, the mice were anesthetized by isoflurane (induction by 4–5%; maintenance by 1–2% in oxygen from a precision vaporizer). Once anesthetized, the mice were placed in the cradle with electrocardiographic electrodes attached via tape to a front and back paw of the mice. To quantify the RV and LV volumes, the EF, and the LV mass, a fast gradient-echo, low-angle shot sequence was performed in the ventricular short-axis with the following parameters: flip angle 20^o^; repetition time, 8.85 ms; echo time, 2.36 ms; matrix, spatial resolution, 0.13 × 0.15 mm. The conventional CMR parameters of RV and LV end-diastolic volume, the LV mass, the RV and LV EF were analyzed on the fast gradient-echo sequence using commercially available software, CVI 42 (Calgary, CA). To assess the myocardial edema, T2-relaxation time was quantified. A respiratory and electrocardiogram-gated, multiecho, ventricular short-axis, spin-echo sequence was performed with 5 spin-echo times (7.8, 15.6, 23.4, 31.2, 39 and 46.8 ms). The sequence was performed prior to contrast administration with the following parameters: single slice at mid-ventricular level, slice thickness of 1 mm, matrix of 128 × 128, spatial resolution of 0.23 × 0.23 mm, and 2 averages. Triggering was done at usually every second to third cardiac cycle with a repetition time of 200 to 400 ms. To quantify T2-average, the endocardial and epicardial border on the mid-ventricular slice, as it had the best image quality, was traced for all echo times and analyzed using commercially available software, CVI 42 (Calgary, CA). A region of interest was traced in the LV free wall and interventricular septum and averaged together. Myocardial fibrosis was assessed by measuring the extracellular volume fraction (ECV). The ECV was calculated by pre-contrast and post-contrast T1 measurements. For measurement of the T1 time, a gadolinium-based contrast agent (0.2 mmol/kg) was diluted in saline at a 1:10 ratio and given via intraperitoneal injection. Similarly to T2 quantification, the T1 was measured at the LV mid-ventricular level in a short-axis slice, both pre-contrast and at several intervals post-contrast using a Look-Locker technique. The Look-Locker sequence was electrocardiogram-gated with a non-slice selective inversion pulse with the following parameters: gradient-echo readout, flip angle 10^o^, repetition time 2.2 ms, echo time 1.6 ms, in-plane resolution of 0.13 × 0.15 mm, slice thickness of 1 mm, repetition time per segment of 22 ms, and number of averages 6 (pre-contrast) or 4 (post-contrast). The post-contrast sequence was performed every 6–8 min following intraperitoneal contrast injection. A region of interest was drawn in the LV free wall and interventricular septum at each time interval, plotted over time and then averaged together for myocardial T1 time. A similar process was performed for a region of interest in the blood pool for ECV calculation. The ECV was calculated using known hematocrit for the mice strain. The global circumferential and longitudinal strain (GCS and GLS, respectively) were acquired at a mid-ventricular short-axis slice for GCS and a single 4-chamber slice for GLS by using a Complementary Spatial Modulation of Magnetization (CSPAMM) technique. The following parameters were used for each sequence: flip angle 20^o^, repetition time7 ms, echo time 3 ms, in-plane resolution of 0.14 × 0.14 mm, slice thickness of 1 mm. The gridlines were analyzed for global peak strain values using commercially available software, Myocardial Solutions (Morrisville, NC).

### Statistical analysis

The R statistical environment was used to perform a one-way ANOVA followed by a post-hoc analysis using the Tukey HSD test on the CMR parameters in relationship to the treatments. Pearson and Spearman correlations between the CMR parameters at the measured time points were calculated using the R statistical environment. Correlations were considered statistically significant for *p*-values less than 0.05.

### Ventricular dysfunction predictive modeling

A logistic generalized linear model was developed in the R statistical environment to predict ventricular dysfunction. Ventricular dysfunction in our model occurs when the LVEF or RVEF was less than or equal to 50%. The parameters measured at 4 weeks were used to predict dysfunction at either 8 or 12 weeks and the parameters measured at 8 weeks were used to predict dysfunction at only 12 weeks. For a one-parameter model a Chi-squared test was used to determine if the model performed better than a constant intercept only model. When we added a second parameter to the model a Chi-squared test was again used, but we compared the two-parameter model to the following one-parameter models: LVEF, RVEF, GLS or T2 time. Receiver operator curves were generated using the ROCR package in the R statistical environment [18].

## Results

All treatments were administrated without complications. We did not observe any death due to drug toxicity. Mice were euthanized at the end of the study as originally intended.

### Ventricular size and function

**Fig. 1:**
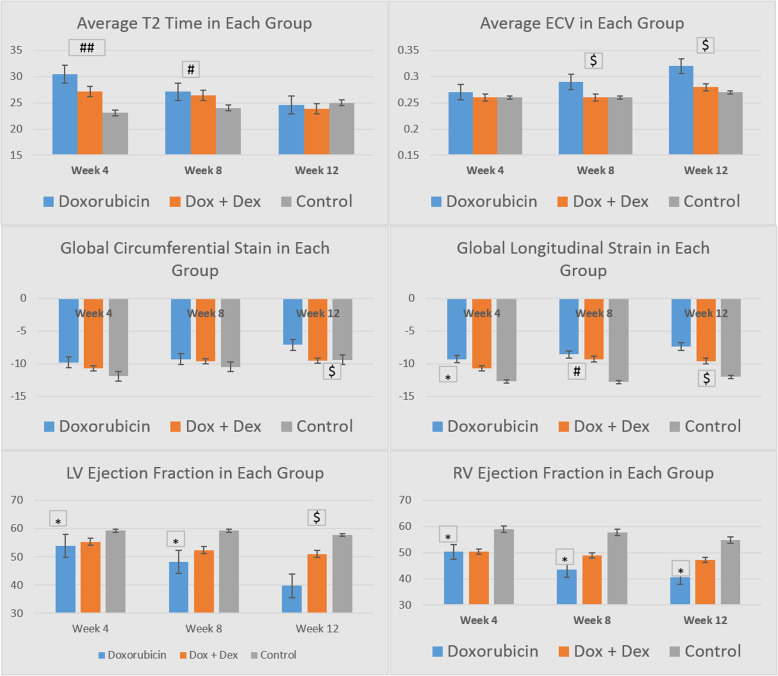
Progression of cardiac magnetic resonance parameters in mice exposed to doxorubicin with and without cardioprotection. We observed main differences at week 12 in ECV, GCS, GLS, LVEF and RVEF between the DOX/DEX (orange bars) and control group (gray bars) compared to the DOX group (blue bars). * indicates an adjusted *p*-value < 0.05 for DOX compared to control group, # indicates an adjusted *p*-value < 0.05 for both DOX and DOX/DEX compared to control group, $ indicates an adjusted *p*-value < 0.05 for DOX compared to both DOX/DEX and control group, ## indicated an adjusted *p*-value < 0.05 for all three groups compared to one another. CMR = cardiac magnetic resonance, Control = saline solution, DOX = doxorubicin, DOX/DEX = doxorubicin plus dexrazoxane, ECV = extracellular volume, GCS = global circumferential strain, GLS = global longitudinal strain, LVEF = left ventricular ejection fraction, RVEF = right ventricular ejection fraction, T2 Avg = T2 average

Cancer therapy-related cardiac dysfunction is characterized by increases in ventricular size and decrease in ventricular function, which were measured by LV volume, LV mass to volume ratio and both right or left EF in our experimental design. All of the mice in the DOX group demonstrated a decrease in LVEF and RVEF to levels below 50% at the week 12 time point. One mouse died after the week 4 time interval. The DOX/DEX group demonstrated 7 of the mice having LVEF below 50% while 12 of the mice had RVEF below 50%. None of the control mice demonstrated LVEF below 50%, and 3 mice demonstrated a RVEF below 50%. Although LV volume significantly increased in all experimental groups, the effect was more noticeable in the DOX and DOX/DEX groups compared to the control group at all evaluated time points (Table 1). Moreover, the ratio of LV mass to volume was decreased in the DOX group compared to the DOX/DEX group, and the LV mass increased in the control and DOX/DEX groups through the time intervals. We also observed a significantly progressive decrease in RVEF and LVEF in the DOX group compared to the other experimental groups (Table 1 and Fig. 1).

**Table 1:** Parameter Difference with Statistical Significance Between Treatments with Time. * Denotes significant difference between DOX and control; # denotes significant difference between DOX and DOX/DEX; $ Denotes significant difference between DOX/DEX and control

Parameter	Average ± Std	Average ± Std	Average ± Std
WEEK 4			
LV Volume (μL)	114.8 ± 8.99*	117.5 ± 7.59**#**	104.83 ± 6.68
LV Mass (μg)	80.07 ± 8.58	86.4 ± 6.82	80.17 ± 7.28
LVEF	53.93 ± 4.01 *	55.3 ± 3.97	59.17 ± 3.37
RVEF	50.27 ± 4.15 *	50.5 ± 2.12**#**	59 ± 2.0
GLS	- 9.29 ± 2.09 *	- 10.73 ± 1.23	−12.72 ± 1.33
T1 Pre (msec)	1283.27 ± 127.96	1288.4 ± 81.86	1261.17 ± 47.27
T1 Post (msec)	576.33 ± 53.31	568.1 ± 52.09	548.17 ± 37.03
ECV	0.27 ± 0.02	0.26 ± 0.01	0.26 ± 0.01
T2 Avg (msec)	30.47 ± 3.38 *	27.2 ± 2.35**#**	23.17 ± 1.47**$**
Mass.Vol	0.7 ± 0.04	0.74 ± 0.08	0.77 ± 0.09
WEEK 8			
LV Volume (μL)	132.8 ± 7.0 *	133.8 ± 6.18**#**	116.67 ± 7.42
LV Mass (μg)	89.53 ± 17.66	102 ± 8.77	93.5 ± 9.09
LVEF	48.2 ± 7.52 *	52.6 ± 3.57	59.17 ± 1.83
RVEF	43.53 ± 9.33 *	49 ± 5.98	57.67 ± 4.13
GLS	- 8.55 ± 1.93 *	- 9.29 ± 1.52**#**	- 12.83 ± 1.12
T1 Pre (msec)	1327.67 ± 85.76	1280.6 ± 83.97	1252.17 ± 58.53
T1 Post (msec)	539.47 ± 43.69	565.9 ± 37.5	565.67 ± 39.07
ECV	0.29 ± 0.03 *	0.26 ± 0.01	0.26 ± 0.01**$**
T2 Avg (msec)	27.13 ± 1.96 *	26.4 ± 1.07**#**	24 ± 1.1
Mass.Vol	0.67 ± 0.12 *	0.76 ± 0.06	0.81 ± 0.1
WEEK 12			
LV Volume (μL)	141.27 ± 7.6 *	139.7 ± 8.55**#**	129.17 ± 3.92
LV Mass (μg)	95.2 ± 24.83	115.5 ± 10.33	101.5 ± 5.54**$**
LVEF	39.67 ± 6.88 *	51 ± 5.37	57.67 ± 3.08**$**
RVEF	40.73 ± 8.63 *	47.2 ± 6.58	54.83 ± 4.07
GLS	- 7.37 ± 1.97 *	- 9.6 ± 1.86	- 12.0 ± 1.78**$**
T1 Pre (msec)	1298.53 ± 77.38	1276.4 ± 77.59	1260.83 ± 43.78
T1 Post (msec)	532.2 ± 50.34	543.3 ± 56.02	566.33 ± 52.99
ECV	0.32 ± 0.04 *	0.28 ± 0.03	0.27 ± 0.02**$**
T2 Avg (msec)	24.6 ± 1.76	23.9 ± 1.1	25.0 ± 1.55
Mass.Vol	0.68 ± 0.19 *	0.83 ± 0.11	0.79 ± 0.06**$**

### Myocardial edema

The presence of myocardial edema, obtained by T2 imaging techniques, may provide evidence of myocardial injury after anthracycline exposure [19]. We observed that mice exposed to doxorubicin with and without dexrazoxane developed myocardial edema compared to untreated mice (control group). This change is characterized by the increase on T2-average imaging at weeks 4 and 8. This effect was more noticeable in the DOX group compared to the DOX/DEX group (Table 1 and Fig. 1), and there was a corresponding statistical difference between the Dox mice and the DOX/DEX mice (Table 1). Interestingly, we did not observe myocardial edema at week 12 in any of the experimental groups (T2-average was similar in the control and both treatment groups). In our experimental design, the CMRs obtained at week 12 were performed 6 weeks after last exposure to doxorubicin.

### Myocardial fibrosis

Extracellular volume expansion is associated with diffuse myocardial fibrosis, a hallmark of pathologic remodeling observed in heart failure and hypertrophic cardiomyopathy [20]. We observed a distinguishable continuous increase in ECV in the DOX group from 4 to 12 weeks. Although, there was a very mild increase in the ECV also in the DOX/DEX group, it was not near the remarkable change observed in the DOX group. In fact, at the later time intervals of week 8 and week 12, there was a statistical difference between the DOX group and the DOX/DEX group in the ECV parameter (Table 1). This statistical difference was also seen when comparing the DOX group with control mice at weeks 8 and 12, but there was not statistical difference between the control group and the DOX/DEX group at any time point.

### Myocardial deformation

**Fig. 2:**
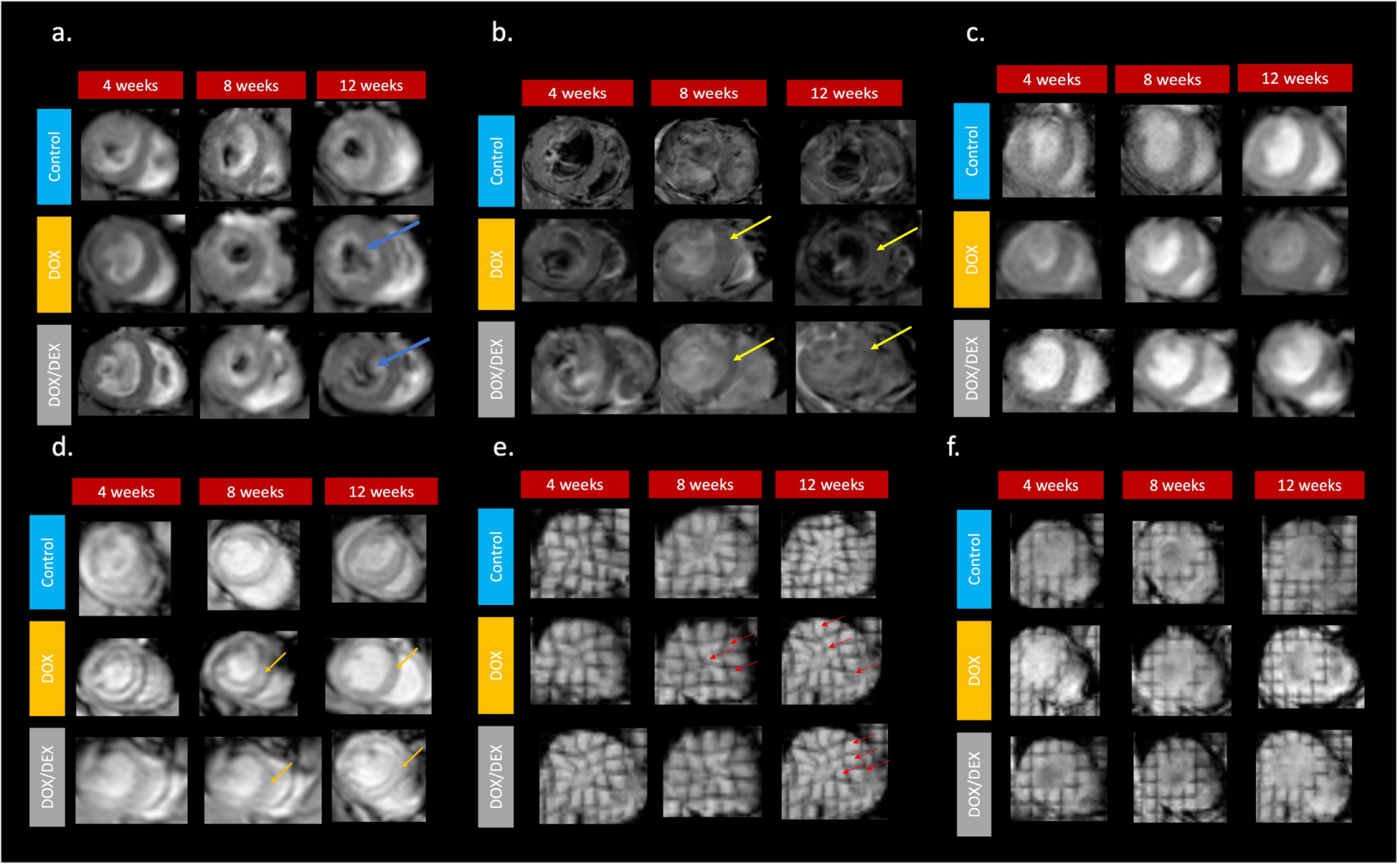
Preclinical Cardiac MRI data. **a** Anatomy scans: We observed that the LV volume was significantly increased in all experimental groups with the effect being more noticeable in the DOX and DOX/DEX groups compared to the control group at all evaluated time points (blue arrows). Upon analysis, the data also show the ratio of LV mass to volume was decreased in the DOX group compared to the DOX/DEX group, and the LV mass was increased in the control and DOX/DEX groups. Please see Table 1 for quantification; **b** T2 Measurement to assess edema: We observed that mice exposed to doxorubicin with and without dexrazoxane developed myocardial edema (yellow arrows) compared to the untreated controls group at both 4 and 8 weeks of age. This effect was more noticeable in the DOX group compared to the DOX/DEX group. Please see Table 1 for quantification; c/d). ECV pre T1 (**c**) and post T1 (**d**) We observed a distinguishable continuous increase in ECV in the DOX group from 4 to 12 weeks. Although an ECV increase was present as well in the DOX/DEX group, it was not as remarkable as the change observed in the DOX group; e/f Tagged scans -- end systole (**e**) and end diastole (**f**). We observed that GLS worsened in the DOX group compared to the other experimental groups as early as 4 weeks. This effect (worsening of GLS) was still observed at 8 weeks in the DOX group, and became evident in the DOX/DEX group. GLS quantification was presented in Fig. 1

Myocardial deformation imaging detects early contractile dysfunction in patients with ischemic heart disease, dilated cardiomyopathy and myocarditis [20]. Global longitudinal strain provides a degree of myocardial deformation of the LV, and it is recommended to be obtained in patients exposed to anthracyclines [21]. We observed that GLS worsened (i.e., became less negative) in the DOX group compared to the other experimental groups as early as 4 weeks. GLS worsening was still observed at 8 weeks in the DOX group, and became evident in the DOX/DEX group. However, impaired GLS was pronounced at 12 weeks only in the DOX group while the DOX/DEX group demonstrated a trend back to normalization of the GLS. Representative CMR images of changes in ventricular size and function, and myocardial edema, fibrosis and deformation in all experimental groups are depicted in Fig. 2.

### Predictive CMR parameters of early cardiac dysfunction

**Fig. 3:**
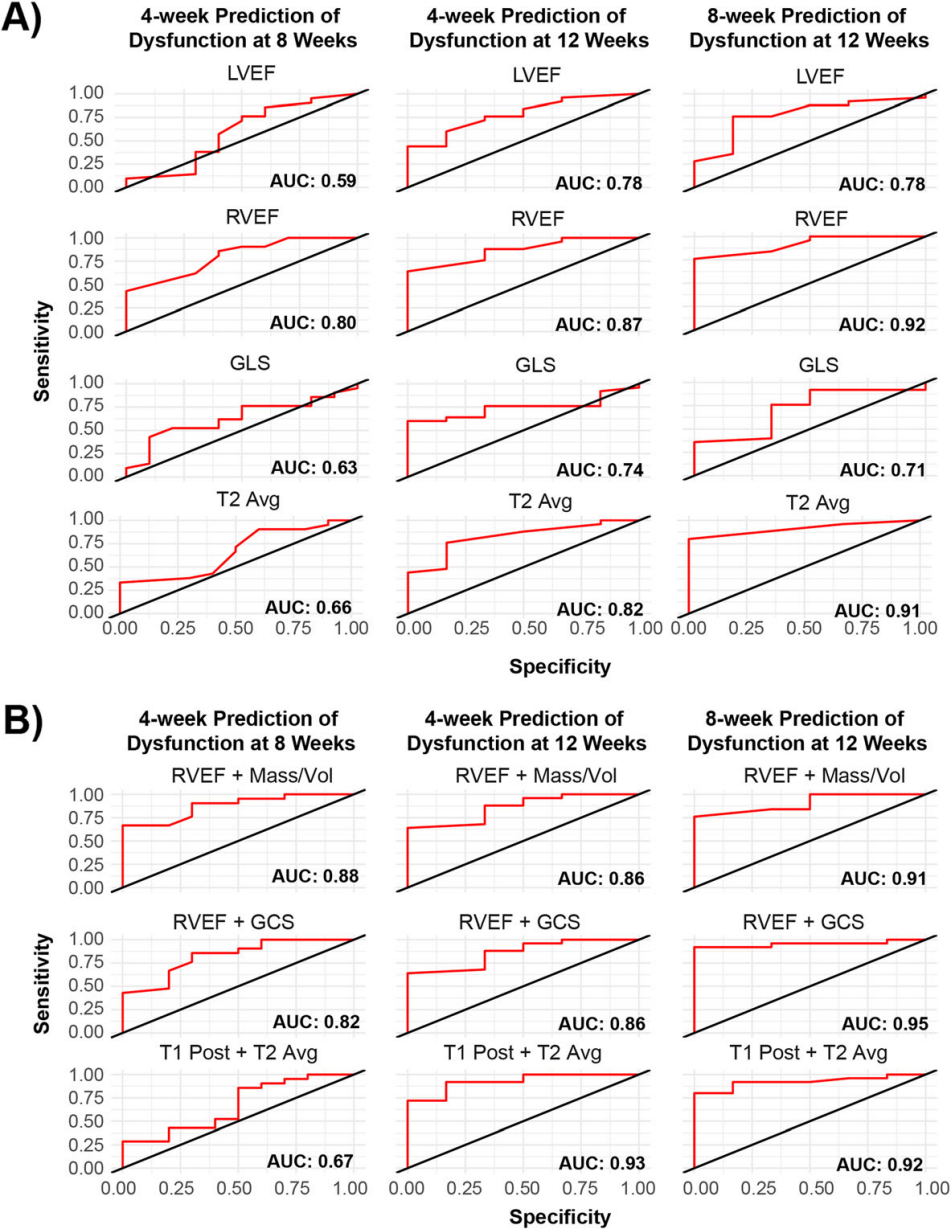
Select receiver operator curves for one-parameter (**A**) and two-parameter (**B**) models predicting ventricular dysfunction. CMR = cardiac magnetic resonance, DOX = doxorubicin, DOX/DEX = doxorubicjn plus dexrazoxane, ECV = extracellular volume, GCS = global circumferential strain, GLS = global longitudinal strain, LVEF = left ventricular ejection fraction, RVEF = right ventricular ejection fraction, T2 Avg = T2 average

The CMR parameters that measured fibrosis (ECV), edema (T2-average) and deformation (GLS) tended to be negatively correlated with ejection fraction, LV mass and LV volume (Supplemental Fig. 1 and Supplemental Fig. 2). We examined the correlation between different CMR parameters measured at 4 weeks and 8 weeks and compared them to future LVEF and RVEF measurements (either at 8 weeks or 12 weeks) for the purpose of gaining insight into their predictive capabilities. The CMR parameters GLS and T2-average in the DOX group at 4 weeks were negatively correlated to 12-week LVEF (Supplemental Fig. 3 and Supplemental Fig. 4). We then explored whether or not the measured CMR parameters at 4 and 8 weeks could predict the future occurrence of ventricular dysfunction, which we defined as LVEF or RVEF less than or equal to 50%. We looked at three criteria when deciding whether or not a one-parameter model was a good predictor of future dysfunction: the coefficient for the tested CMR parameter was statistically significant (*p*-value < 0.05), the *p*-value from a Chi-squared test comparing the one-parameter model to an intercept only model was statistically significant (*p*-value < 0.05), and the area under the curve (AUC) from a receiver operator curve was greater than 0.70. The AUC measures how well a model classifies different states. An AUC of 0.5 indicates that the models has a 50% chance of predicting two different states while and AUC of 1.0 indicates that the model is perfectly accurate. The parameters LVEF, RVEF, GLS and T2-average were good predictors of dysfunction at 12 weeks when the parameters were measured at both 4 and 8 weeks (Fig. 3, and Supplemental Figs. 3 and 4).

The same criteria was applied to the development of a two-parameter model with the following adjustments: the coefficient for each CMR parameter in the model was statistically significant (*p*-value < 0.05), the *p*-values for the Chi-squared test comparing the two-parameter to the one-parameter models RVEF, LVEF, GLS and T2-average were all statistically significant (*p*-value < 0.05), and the AUC from the receiver operator curve was greater than 0.70. Using this criteria, we found that the model combining the CMR parameters T1-post plus T2- average was a good predictor of dysfunction at 12 weeks when the parameters were measured at 4 weeks (Fig. 3, and Supplemental Figs. 3 and 4).

## Discussion

We demonstrated in a pre-clinical mouse model of CTRCD using CMR that myocardial edema is associated with myocardial deformation abnormalities prior to the development of myocardial fibrosis and global biventricular dysfunction. Moreover, the use of dexrazoxane did not completely prevent the initial myocardium injury (T2-average) by doxorubicin, but it did limit the degree of edema formation and perhaps more importantly, it prevented the chronic inflammation characterized by persistent fibrosis (ECV) and worsening contractile function (GLS).

We observed the early development of myocardial edema in mice exposed to anthracycline. This is similar to the results obtained by Farhad et al., and Galan-Arriola et al., that also observed the appearance of myocardial edema in different animal models using CMR imaging after doxorubicin treatment [15, 16]. However, our study included an experimental group that received cardioprotection with dexrazoxane. In this cardioprotection group, the T2-average was elevated at the initial 4-week time point compared to controls, but not to the extent of the group that received only doxorubicin. While dexrazoxane has been shown to decrease the incidence of left ventricular dysfunction, the exact mechanism is not fully understood [7]. This finding of abnormal T2-relaxation time, consistent with myocardial edema but not the later development of ventricular dysfunction or myocardial fibrosis, suggests that cardioprotection with dexrazoxane may not fully prevent the initial cardiomyocyte swelling but aids in myocardial tissue recovery. We speculate that this effect may be caused by the reduction in the production of free oxygen radicals in the myocardium. Essentially, while anthracyclines still induce inflammation and myocardial edema, the cardioprotection with dexrazoxane appears to augment myocardial recovery. Our pre-clinical model results also suggest that the quantification of myocardial edema during anthracycline administration could be investigated as an early marker of ventricular dysfunction in patients undergoing cancer treatment.

Similar to the previous study by Farhad et al., we observed a continuous expansion of the ECV in the anthracycline group. However, our study distinctly demonstrated that the cardioprotection group, as well as the control group, did not have an expansion in the ECV. This result indicates the cardioprotective effect of dexrazoxane in the myocardium attenuates the development of myocardial fibrosis. The ECV expansion demonstrated a negative correlation with LVEF and to predict an increase in cardiovascular morbidity and mortality in large adult studies [21, 22]. Pediatric cancer survivors exposed to anthracyclines with findings of myocardial fibrosis, measured by ECV expansion in our study, have higher rates of hospitalizations due to heart failure [19]. We encountered that the combination of post-contrast T1 and T2 values was a good predictor of ventricular dysfunction in mice exposed to doxorubicin. The utilization of multiple imaging parameters for prediction of cardiovascular disease would ultimately lead to improved clinical care and to tailor cancer treatment to the individual based upon their cardiac risk. We believe that prior studies and our data would support routine quantification of the ECV using CMR in long-term cancer survivors as standard of care considering its power in predicting an increase in cardiovascular morbidity and mortality. Due to the strong correlation of ECV with cardiac dysfunction, abnormalities in the ECV may warrant closer investigation while normal values of the ECV may allow longer surveillance intervals in the long-term survivors.

Another unique feature of our model compared to previous pre-clinical studies was the inclusion of myocardial deformation. We observed worsening of the GLS at the earliest CMR time interval (several weeks prior to the development of global ventricular dysfunction) in the experimental group that received doxorubicin. Nevertheless, cardioprotection with dexrazoxane appeared to somewhat attenuate that effect as there was no difference at the 4 week time interval between the cardioprotection group and the control group. However, this finding did not hold at the later time interval of 8 weeks. Deformation imaging by TTE has become more prevalent in its use for detecting cardiac dysfunction in patients receiving cancer treatment [23, 24]. Similar to myocardial edema, few studies have been performed to examine the ability of myocardial strain to predict a decrease in global systolic function and the majority of studies examining deformation have utilized echocardiography [25–27]. Myocardial deformation analysis could be a key component in the evaluation of anthracycline-induced CTRCD with more extended use of CMR imaging. The changes observed in GLS after a longitudinal follow-up in our animal study would support that recommendation.

Cardiac magnetic resonance imaging is the reference standard to measure cardiac volume and mass, and thus is optimally suited to characterize the cardiac remodeling process. Our study demonstrated an expansion of the LV volume with a relative decrease in the LV mass to volume ratio. This finding was seen only in the group treated with doxorubicin compared to the cardioprotection and control groups. This finding continues to support the observation that initial myocardial edema may be present even with the use of dexrazoxane, but expansion of the LV volume and “thinning” of the LV wall do not occur. The expansion of the LV volume is more related with the development of dilated cardiomyopathy, as it has previously been reported in pediatric cancer survivors [7, 28]. We observe that our results contrasts with the Grinch Syndrome model, where there is both a decrease in LV volume and LV mass, and this may be a reflection of the relative time periods of the cardiac development [29]. Although we did not evaluate diastolic function, our CMR findings warrant further clinical investigation using this imaging modality to fully characterize the progression of cardiac remodeling after anthracycline exposure.

Cardiac magnetic resonance has become the imaging reference standard to evaluate for ventricular volume, ventricular mass and EF in different cardiac diseases, and provides better characterization of the myocardium [13]. Our study demonstrated the value of both myocardial edema and myocardial deformation using CMR to predict the development of LV systolic function. Moreover, the use of dexrazoxane does not appear to prevent the initial cardiac injury after anthracycline treatment but promotes a rapid myocardium repair. We believe that evaluation of myocardial edema and myocardial deformation to predict LV dysfunction in patients treated with anthracyclines, as well the immediate effect of dexrazoxane in the myocardium by CMR merits further evaluation in a clinical trial. The CMR would certainly not be a replacement of TTE, but could be employed in patients that require a more comprehensive myocardial characterization [30, 31]. The potential to monitor multiple CMR parameters during cancer treatment to allow possible modification of therapies would have tremendous clinical benefit, and potentially decrease the incidence of CTRCD. We consider that these observations should be investigated by using CMR both during select intervals of anthracycline administration, as well as during long-term follow up.

### Limitations

The main limitation of our study is the use of a mouse model. There is significant variability using animal models to study the effect of anthracycline on cardiac remodeling, and thus our results may be relevant only to this mouse model. Nevertheless, we consider that our findings increase our knowledge about CTRCD and our results should be evaluated in patients exposed to anthracyclines. Another limitation is the lack of correlative histology evaluation of chronic inflammation. However, ECV have been closely correlated with myocardial fibrosis in previous studies [14, 15].

## Conclusion

T2 time and deformation by CMR appears to identify early myocardial injury from anthracyclines. Dexrazoxne did not seem to prevent the initial edema as gauged by T2 time, but the inflammatory changes were not sustained. CMR may be useful for early detection of cardiac dysfunction. Serial CMR demonstrates dexrazoxane minimizes cardiac dysfunction and aids recovery in a mouse model.

## Supplementary Information

**Additional file 1: Supplemental Table 1.** Correlation Between Parameters as Time Progresses in Control Samples.

**Additional file 2: Supplemental Table 2.** Correlation Between Parameters as Time Progresses in Doxorubicin Samples.

**Additional file 3: Supplemental Table 3.** Correlation Between Parameters as Time Progresses in Doxorubicin plus Dexrazoxane Samples.

**Additional file 4: Supplemental Table 4.** Two Parameter Model Performance.

**Additional file 5.**

**Additional file 6.**

**Additional file 7.**

**Additional file 8.**

## Data Availability

The datasets during and/or analyzed during the current study available from the corresponding author on reasonable request.

## Abbreviations

CTRCD: CANCER therapy-related cardiac dysfunction
EF: Ejection Fraction
TTE: Trans-thoracic echocardiography
LVSF: Left ventricle shortening fraction
LVEF: Left ventricle ejection fraction
CMR: Cardiac magnetic resonance
ECV: Extracellular volume
DOX: Doxorubicin
DOX/DEX: Doxorubicin/dexrazoxane
GLS: Global longitudinal strain

## References

[1] Norris RF, Adamson PC (2012). Challenges and opportunities in childhood cancer drug development. Nat Rev Cancer.

[2] Lipshultz SE, Sambatakos P, Maguire (2014). Cardiotoxicity and cardioprotection in childhood cancer. Acta Haematol.

[3] Yeh ET, Bickford CL (2009). Cardiovascular complications of cancer therapy: incidence, pathogenesis, diagnosis, and management. J Am Coll Cardiol.

[4] Mulrooney DA, Yeazel MW, Kawashima T, Mertens AC, Mitby P, Stovall M, Donaldson SS, Green DM, Sklar CA, Robison LL, Leisenring WM (2009). Cardiac outcomes in a cohort of adult survivors of childhood and adolescent cancer: retrospective analysis of the childhood Cancer survivor study cohort. BMJ.

[5] Mertens AC, Liu Q, Neglia JP, Wasilewski K, Leisenring W, Armstrong GT, Robison LL, Yasui Y (2008). Cause-specific late mortality among 5-year survivors of childhood cancer: the childhood Cancer survivor study. J Natl Cancer Inst.

[6] Lipshultz SE, Scully RE, Lipsitz SR, Sallan SE, Silverman LB, Miller TL, Barry EV, Asselin BL, Athale U, Clavell LA, Larsen E, Moghrabi A, Samson Y, Michon B, Schorin MA, Cohen HJ, Neuberg DS, Orav EJ, Colan SD (2010). Assessment of dexrazoxane as a cardioprotectant in doxorubicin-treated children with high-risk acute lymphoblastic leukaemia: long-term follow-up of a prospective, randomised, multicentre trial. Lancet Oncol.

[7] Lipshultz SE, Cochran TR, Franco VI, Miller TL (2013). Treatment-related cardiotoxicity in survivors of childhood cancer. Nat Rev Clin Oncol.

[8] Getz KD, Sung L, Alonzo T (2020). Effect of Dexrazoxane on left ventricular systolic function and treatment outcomes in patients with acute myeloid leukemia: a report from the Children's oncology group. J Clin Oncol.

[9] Van der Pal HJ, van Dalen EC, Hauptmann M (2010). Cardiac function in 5-year survivors of childhood cancer: a long-term follow-up study. Arch Intern Med.

[10] Armstrong GT, Plana JC, Zhang N, Srivastava D, Green DM, Ness KK, Daniel Donovan F, Metzger ML, Arevalo A, Durand JB, Joshi V, Hudson MM, Robison LL, Flamm SD (2012). Screening adult survivors of childhood cancer for cardiomyopathy: comparison of echocardiography and cardiac magnetic resonance imaging. J Clin Oncol.

[11] Zamorano JL, Lancellotti P, Rodriguez Muñoz D, Aboyans V, Asteggiano R, Galderisi M, Habib G, Lenihan DJ, Lip GYH, Lyon AR, Lopez Fernandez T, Mohty D, Piepoli MF, Tamargo J, Torbicki A, Suter TM, Zamorano JL, Aboyans V, Achenbach S, Agewall S, Badimon L, Barón-Esquivias G, Baumgartner H, Bax JJ, Bueno H, Carerj S, Dean V, Erol Ç, Fitzsimons D, Gaemperli O, Kirchhof P, Kolh P, Lancellotti P, Lip GYH, Nihoyannopoulos P, Piepoli MF, Ponikowski P, Roffi M, Torbicki A, Vaz Carneiro A, Windecker S, Achenbach S, Minotti G, Agewall S, Badimon L, Bueno H, Cardinale D, Carerj S, Curigliano G, de Azambuja E, Dent S, Erol C, Ewer MS, Farmakis D, Fietkau R, Fitzsimons D, Gaemperli O, Kirchhof P, Kohl P, McGale P, Ponikowski P, Ringwald J, Roffi M, Schulz-Menger J, Stebbing J, Steiner RK, Szmit S, Vaz Carneiro A, Windecker S, Authors/Task Force Members, ESC Committee for Practice Guidelines (CPG), Document Reviewers (2017). 2016 ESC position paper on cancer treatments and cardiovascular toxicity developed under the auspices of the ESC Committee for practice guidelines: the task force for cancer treatments and cardiovascular toxicity of the European Society of Cardiology (ESC). Eur J Heart Fail.

[12] Grothues F, Smith GC, Moon JC (2002). Comparison of interstudy reproducibility of cardiovascular magnetic resonance with two-dimensional echocardiography in normal subjects and in patients with heart failure or left ventricular hypertrophy. Am J Cardiol.

[13] Messroghli DR, Moon JC, Ferreira VM (2017). Clinical recommendations for cardiovascular magnetic resonance mapping of T1, T2, T2* and extracellular volume: a consensus statement by the Society for Cardiovascular Magnetic Resonance (SCMR) endorsed by the European Association for Cardiovascular Imaging (EACVI). J Cardiovasc Magn Reson.

[14] Haaf P, Garg P, Messroghli DR (2016). Cardiac T1 mapping and extracellular volume (ECV) in clinical practice: a comprehensive review. J Cardiovasc Magn Reson.

[15] Galán-Arriola C, Lobo M, Vílchez-Tschischke JP (2019). Serial Magnetic Resonance Imaging to Identify Early Stages of Anthracycline-Induced Cardiotoxicity. J Am Coll Cardiol.

[16] Farhad H, Staziaki PV, Addison D (2016). Characterization of the changes in cardiac structure and function in mice treated with anthracyclines using serial cardiac magnetic resonance imaging. Circ Cardiovasc Imaging.

[17] Neilan TG, Coelho-Filho OR, Shah RV, Abbasi SA, Heydari B, Watanabe E, Chen Y, Mandry D, Pierre-Mongeon F, Blankstein R, Kwong RY, Jerosch-Herold M (2013). Myocardial extracellular volume fraction from T1 measurements in healthy volunteers and mice: relationship to aging and cardiac dimensions. JACC Cardiovasc Imaging.

[18] Sing T, Sander O, Beerenwinkel N, Lengauer T (2005). ROCR: visualizing classifier performance in R. Bioinformatics..

[19] Neilan TG, Coelho-Filho OR, Shah RV, Feng JH, Pena-Herrera D, Mandry D, Pierre-Mongeon F, Heydari B, Francis SA, Moslehi J, Kwong RY, Jerosch-Herold M (2013). Myocardial extracellular volume by cardiac magnetic resonance imaging in patients treated with anthracycline-based chemotherapy. Am J Cardiol.

[20] Kammerlander AA, Marzluf BA, Zotter-Tufaro C, Aschauer S, Duca F, Bachmann A, Knechtelsdorfer K, Wiesinger M, Pfaffenberger S, Greiser A, Lang IM, Bonderman D, Mascherbauer J (2016). T1 mapping by CMR imaging: from histological validation to clinical implication. JACC Cardiovasc Imaging.

[21] Wong TC, Piehler K, Meier CG, Testa SM, Klock AM, Aneizi AA, et al. Association between extracellular matrix expansion quantified by cardiovascular magnetic resonance and short-term mortality. Circulation. 2012;126(10):1206–16. 10.1161/CIRCULATIONAHA.111.089409. 10.1161/CIRCULATIONAHA.111.089409 PMC3464491 22851543

[22] Schelbert EB, Piehler KM, Zareba KM (2015). Myocardial fibrosis quantified by extracellular volume is associated with subsequent hospitalization for heart failure, death, or both across the Spectrum of ejection fraction and heart failure stage. J Am Heart Assoc.

[23] Muser D, Castro SA, Santangeli P, Nucifora G (2018). Clinical applications of feature-tracking cardiac magnetic resonance imaging. World J Cardiol.

[24] Thavendiranathan P, Poulin F, Lim KD, Plana JC, Woo A, Marwick TH (2014). Use of myocardial strain imaging by echocardiography for the early detection of cardiotoxicity in patients during and after cancer chemotherapy: a systematic review. J Am Coll Cardiol.

[25] Pignatelli RH, Ghazi P, Reddy SC (2015). Abnormal myocardial strain indices in children receiving anthracycline chemotherapy. Pediatr Cardiol.

[26] Negishi K, Negishi T, Haluska BA, Hare JL, Plana JC, Marwick TH (2014). Use of speckle strain to assess left ventricular responses to cardiotoxic chemotherapy and cardioprotection. Eur Heart J Cardiovasc Imaging.

[27] Poterucha JT, Kutty S, Lindquist RK, Li L, Eidem BW (2012). Changes in left ventricular longitudinal strain with anthracycline chemotherapy in adolescents precede subsequent decreased left ventricular ejection fraction. J Am Soc Echocardiogr.

[28] Toro-Salazar OH, Gillan E, O'Loughlin MT (2013). Occult cardiotoxicity in childhood cancer survivors exposed to anthracycline therapy. Circ Cardiovasc Imaging.

[29] Lipshultz SE, Scully RE, Stevenson KE, Franco VI, Neuberg DS, Colan SD, Silverman LB, Moslehi JJ, Cheng S, Sallan SE (2014). Hearts too small for body size after doxorubicin for childhood ALL: Grinch syndrome. J Clin Oncol.

[30] Meyersohn NM, Pursnani A, Neilan TG (2015). Detection of cardiac toxicity due to Cancer treatment: role of cardiac MRI. Curr Treat Options Cardiovasc Med.

[31] Lopez-Fernandez T, Thavendiranathan P (2017). Emerging cardiac imaging modalities for the early detection of cardiotoxicity due to anticancer therapies. Rev Esp Cardiol.

