# Supplementary material for: Early detection of myocardial changes with and without dexrazoxane using serial magnetic resonance imaging in a pre-clinical mouse model
Source: Cardiooncology. 2021 Jun 16;7:23. doi: 10.1186/s40959-021-00109-8 (PMC8207719; doi:10.1186/s40959-021-00109-8)
Supplement: Supplementary file 3 — Additional file 3: Supplemental Table 3. Correlation Between Parameters as Time Progresses in Doxorubicin plus Dexrazoxane Samples. [file 40959_2021_109_MOESM3_ESM.docx]

| Supplemental Table 3: Correlation Between Parameters as Time Progresses in Doxorubicin plus Dexrazoxane Samples | | | | | | | |
| --- | --- | --- | --- | --- | --- | --- | --- |
|  |  | **Pearson** | | | | | |
|  |  | **4 weeks** | | **8 weeks** | | **12 weeks** | |
| **Parameter 1** | **Parameter 2** | **Correlation** | **p-value** | **Correlation** | **p-value** | **Correlation** | **p-value** |
| Volume | Mass | -0.09 | 0.81 | 0.44 | 0.20 | -0.73 | 0.02 |
| Volume | LVEF | 0.20 | 0.58 | -0.50 | 0.14 | -0.93 | 0.00 |
| Mass | LVEF | -0.70 | 0.02 | 0.04 | 0.91 | 0.77 | 0.01 |
| Volume | RVEF | 0.13 | 0.73 | -0.16 | 0.67 | -0.75 | 0.01 |
| Mass | RVEF | -0.12 | 0.74 | 0.33 | 0.35 | 0.55 | 0.10 |
| LVEF | RVEF | 0.51 | 0.13 | 0.40 | 0.25 | 0.84 | 0.00 |
| Volume | GCS | 0.51 | 0.13 | 0.43 | 0.21 | 0.85 | 0.00 |
| Mass | GCS | -0.44 | 0.20 | 0.32 | 0.37 | -0.84 | 0.00 |
| LVEF | GCS | 0.14 | 0.69 | -0.24 | 0.50 | -0.82 | 0.00 |
| RVEF | GCS | -0.24 | 0.50 | 0.13 | 0.71 | -0.65 | 0.04 |
| Volume | GLS | -0.42 | 0.23 | 0.23 | 0.52 | 0.71 | 0.02 |
| Mass | GLS | 0.74 | 0.01 | -0.41 | 0.24 | -0.75 | 0.01 |
| LVEF | GLS | -0.58 | 0.08 | -0.13 | 0.72 | -0.75 | 0.01 |
| RVEF | GLS | -0.50 | 0.14 | -0.40 | 0.25 | -0.71 | 0.02 |
| GCS | GLS | -0.28 | 0.43 | 0.24 | 0.50 | 0.87 | 0.00 |
| Volume | T1 Pre | 0.08 | 0.83 | 0.09 | 0.81 | -0.34 | 0.34 |
| Mass | T1 Pre | 0.63 | 0.05 | -0.09 | 0.81 | -0.02 | 0.96 |
| LVEF | T1 Pre | -0.62 | 0.06 | -0.10 | 0.78 | 0.22 | 0.54 |
| RVEF | T1 Pre | -0.38 | 0.28 | 0.48 | 0.16 | 0.06 | 0.86 |
| GCS | T1 Pre | -0.10 | 0.79 | 0.56 | 0.09 | -0.22 | 0.54 |
| GLS | T1 Pre | 0.54 | 0.11 | -0.14 | 0.70 | 0.14 | 0.70 |
| Volume | T1 Post | 0.32 | 0.36 | 0.52 | 0.12 | -0.14 | 0.69 |
| Mass | T1 Post | -0.17 | 0.65 | 0.53 | 0.11 | 0.02 | 0.95 |
| LVEF | T1 Post | -0.32 | 0.37 | -0.07 | 0.86 | 0.22 | 0.54 |
| RVEF | T1 Post | -0.13 | 0.73 | 0.67 | 0.03 | 0.65 | 0.04 |
| GCS | T1 Post | 0.65 | 0.04 | 0.39 | 0.27 | -0.21 | 0.55 |
| GLS | T1 Post | -0.30 | 0.41 | -0.42 | 0.22 | -0.47 | 0.17 |
| T1 Pre | T1 Post | 0.30 | 0.40 | 0.60 | 0.06 | -0.23 | 0.53 |
| Volume | ECV | -0.08 | 0.82 | 0.69 | 0.03 | 0.69 | 0.03 |
| Mass | ECV | 0.56 | 0.09 | -0.08 | 0.82 | -0.91 | 0.00 |
| LVEF | ECV | 0.00 | 0.99 | -0.77 | 0.01 | -0.81 | 0.00 |
| RVEF | ECV | 0.38 | 0.27 | -0.30 | 0.40 | -0.73 | 0.02 |
| GCS | ECV | -0.35 | 0.32 | 0.58 | 0.08 | 0.82 | 0.00 |
| GLS | ECV | 0.35 | 0.32 | 0.59 | 0.07 | 0.77 | 0.01 |
| T1 Pre | ECV | -0.03 | 0.94 | 0.30 | 0.40 | -0.10 | 0.79 |
| T1 Post | ECV | -0.55 | 0.10 | 0.16 | 0.67 | -0.29 | 0.41 |
| Volume | T2 Avg | -0.05 | 0.89 | -0.79 | 0.01 | 0.47 | 0.17 |
| Mass | T2 Avg | 0.84 | 0.00 | -0.02 | 0.95 | -0.52 | 0.12 |
| LVEF | T2 Avg | -0.67 | 0.03 | 0.22 | 0.54 | -0.54 | 0.10 |
| RVEF | T2 Avg | -0.04 | 0.90 | 0.10 | 0.78 | -0.76 | 0.01 |
| GCS | T2 Avg | -0.40 | 0.26 | -0.43 | 0.22 | 0.50 | 0.15 |
| GLS | T2 Avg | 0.49 | 0.15 | -0.38 | 0.28 | 0.72 | 0.02 |
| T1 Pre | T2 Avg | 0.29 | 0.42 | -0.35 | 0.32 | 0.52 | 0.12 |
| T1 Post | T2 Avg | -0.20 | 0.57 | -0.47 | 0.17 | -0.72 | 0.02 |
| ECV | T2 Avg | 0.64 | 0.05 | -0.64 | 0.05 | 0.58 | 0.08 |
| Volume | Mass/Vol | -0.68 | 0.03 | -0.11 | 0.75 | -0.90 | 0.00 |
| Mass | Mass/Vol | 0.78 | 0.01 | 0.84 | 0.00 | 0.95 | 0.00 |
| LVEF | Mass/Vol | -0.65 | 0.04 | 0.32 | 0.36 | 0.90 | 0.00 |
| RVEF | Mass/Vol | -0.17 | 0.65 | 0.45 | 0.20 | 0.67 | 0.03 |
| GCS | Mass/Vol | -0.67 | 0.04 | 0.08 | 0.82 | -0.89 | 0.00 |
| GLS | Mass/Vol | 0.79 | 0.01 | -0.59 | 0.07 | -0.78 | 0.01 |
| T1 Pre | Mass/Vol | 0.42 | 0.23 | -0.17 | 0.64 | 0.12 | 0.73 |
| T1 Post | Mass/Vol | -0.32 | 0.37 | 0.26 | 0.46 | 0.05 | 0.88 |
| ECV | Mass/Vol | 0.44 | 0.21 | -0.50 | 0.14 | -0.87 | 0.00 |
| T2 Avg | Mass/Vol | 0.64 | 0.05 | 0.46 | 0.18 | -0.53 | 0.12 |
|  |  | **Spearman** | | | | | |
| Volume | Mass | 0.15 | 0.68 | 0.55 | 0.10 | -0.49 | 0.15 |
| Volume | LVEF | 0.10 | 0.79 | -0.57 | 0.09 | -0.94 | 0.00 |
| Mass | LVEF | -0.67 | 0.03 | -0.05 | 0.89 | 0.64 | 0.05 |
| Volume | RVEF | 0.04 | 0.90 | -0.20 | 0.58 | -0.55 | 0.10 |
| Mass | RVEF | -0.23 | 0.53 | 0.35 | 0.32 | 0.42 | 0.23 |
| LVEF | RVEF | 0.71 | 0.02 | 0.25 | 0.49 | 0.64 | 0.04 |
| Volume | GCS | 0.41 | 0.24 | 0.26 | 0.47 | 0.64 | 0.05 |
| Mass | GCS | -0.17 | 0.63 | 0.17 | 0.63 | -0.70 | 0.02 |
| LVEF | GCS | -0.21 | 0.56 | -0.23 | 0.52 | -0.65 | 0.04 |
| RVEF | GCS | -0.34 | 0.34 | 0.17 | 0.63 | -0.25 | 0.49 |
| Volume | GLS | -0.26 | 0.47 | 0.18 | 0.62 | 0.63 | 0.05 |
| Mass | GLS | 0.65 | 0.04 | -0.30 | 0.40 | -0.58 | 0.08 |
| LVEF | GLS | -0.65 | 0.04 | 0.02 | 0.95 | -0.73 | 0.02 |
| RVEF | GLS | -0.58 | 0.08 | -0.15 | 0.68 | -0.67 | 0.03 |
| GCS | GLS | -0.09 | 0.80 | 0.40 | 0.26 | 0.75 | 0.01 |
| Volume | T1 Pre | 0.16 | 0.66 | 0.10 | 0.77 | -0.26 | 0.47 |
| Mass | T1 Pre | 0.74 | 0.01 | 0.22 | 0.54 | -0.04 | 0.92 |
| LVEF | T1 Pre | -0.73 | 0.02 | -0.17 | 0.64 | 0.07 | 0.85 |
| RVEF | T1 Pre | -0.61 | 0.06 | 0.53 | 0.11 | -0.15 | 0.68 |
| GCS | T1 Pre | 0.18 | 0.62 | 0.56 | 0.09 | -0.02 | 0.96 |
| GLS | T1 Pre | 0.56 | 0.09 | -0.28 | 0.43 | 0.28 | 0.43 |
| Volume | T1 Post | 0.31 | 0.38 | 0.52 | 0.12 | 0.01 | 0.98 |
| Mass | T1 Post | 0.02 | 0.95 | 0.69 | 0.03 | -0.01 | 0.99 |
| LVEF | T1 Post | -0.38 | 0.28 | -0.26 | 0.46 | 0.06 | 0.87 |
| RVEF | T1 Post | -0.24 | 0.51 | 0.58 | 0.08 | 0.74 | 0.01 |
| GCS | T1 Post | 0.79 | 0.01 | 0.37 | 0.30 | 0.17 | 0.64 |
| GLS | T1 Post | -0.19 | 0.61 | -0.29 | 0.42 | -0.37 | 0.30 |
| T1 Pre | T1 Post | 0.40 | 0.25 | 0.76 | 0.01 | -0.12 | 0.75 |
| Volume | ECV | -0.06 | 0.86 | 0.62 | 0.06 | 0.52 | 0.13 |
| Mass | ECV | 0.46 | 0.18 | -0.01 | 0.97 | -0.96 | 0.00 |
| LVEF | ECV | 0.07 | 0.85 | -0.68 | 0.03 | -0.72 | 0.02 |
| RVEF | ECV | 0.32 | 0.38 | -0.10 | 0.78 | -0.53 | 0.11 |
| GCS | ECV | -0.30 | 0.40 | 0.68 | 0.03 | 0.64 | 0.05 |
| GLS | ECV | 0.22 | 0.55 | 0.57 | 0.09 | 0.65 | 0.04 |
| T1 Pre | ECV | 0.01 | 0.97 | 0.29 | 0.41 | 0.15 | 0.69 |
| T1 Post | ECV | -0.47 | 0.17 | 0.28 | 0.44 | -0.09 | 0.81 |
| Volume | T2 Avg | -0.01 | 0.99 | -0.74 | 0.01 | 0.28 | 0.43 |
| Mass | T2 Avg | 0.82 | 0.00 | -0.21 | 0.57 | -0.47 | 0.17 |
| LVEF | T2 Avg | -0.63 | 0.05 | 0.32 | 0.37 | -0.45 | 0.19 |
| RVEF | T2 Avg | -0.17 | 0.65 | 0.03 | 0.94 | -0.83 | 0.00 |
| GCS | T2 Avg | -0.16 | 0.66 | -0.45 | 0.20 | 0.30 | 0.41 |
| GLS | T2 Avg | 0.47 | 0.17 | -0.41 | 0.24 | 0.67 | 0.03 |
| T1 Pre | T2 Avg | 0.46 | 0.18 | -0.36 | 0.30 | 0.62 | 0.05 |
| T1 Post | T2 Avg | -0.09 | 0.81 | -0.56 | 0.09 | -0.60 | 0.07 |
| ECV | T2 Avg | 0.60 | 0.07 | -0.69 | 0.03 | 0.56 | 0.09 |
| Volume | Mass/Vol | -0.62 | 0.06 | 0.02 | 0.96 | -0.86 | 0.00 |
| Mass | Mass/Vol | 0.60 | 0.07 | 0.80 | 0.01 | 0.81 | 0.00 |
| LVEF | Mass/Vol | -0.53 | 0.11 | 0.27 | 0.45 | 0.90 | 0.00 |
| RVEF | Mass/Vol | -0.28 | 0.43 | 0.45 | 0.20 | 0.54 | 0.11 |
| GCS | Mass/Vol | -0.33 | 0.36 | 0.02 | 0.95 | -0.70 | 0.02 |
| GLS | Mass/Vol | 0.80 | 0.01 | -0.30 | 0.39 | -0.65 | 0.04 |
| T1 Pre | Mass/Vol | 0.36 | 0.31 | -0.02 | 0.95 | 0.03 | 0.93 |
| T1 Post | Mass/Vol | -0.31 | 0.38 | 0.29 | 0.42 | -0.06 | 0.87 |
| ECV | Mass/Vol | 0.53 | 0.11 | -0.37 | 0.29 | -0.78 | 0.01 |
| T2 Avg | Mass/Vol | 0.62 | 0.05 | 0.36 | 0.31 | -0.47 | 0.17 |
