# Supplementary material for: Early detection of myocardial changes with and without dexrazoxane using serial magnetic resonance imaging in a pre-clinical mouse model
Source: Cardiooncology. 2021 Jun 16;7:23. doi: 10.1186/s40959-021-00109-8 (PMC8207719; doi:10.1186/s40959-021-00109-8)
Supplement: Supplementary file 4 — Additional file 4: Supplemental Table 4. Two Parameter Model Performance. [file 40959_2021_109_MOESM4_ESM.docx]

| Supplemental Table 4: Two Parameter Model Performance | | | | | | | | |
| --- | --- | --- | --- | --- | --- | --- | --- | --- |
|  | **4-week prediction of Dysfunction (8 week)** | | | | | | | |
| **Parameter** | **Pr(>\|z\|)** | **Pr(>\|z\|)** | **AIC** | **Chisq**  **(LVEF)** | **Chisq**  **(RVEF)** | **Chisq**  **(GLS)** | **Chisq**  **(T2 Avg)** | **AUC** |
| Volume+Mass | 0.63 | 0.05 | 38.82 | 0.02 | 1.00 | 0.03 | 0.06 | 0.72 |
| Volume+LVEF | 0.56 | 0.21 | 42.75 | 0.20 | 1.00 | 0.34 | 1.00 | 0.67 |
| Volume+RVEF | 0.21 | 0.82 | 35.11 | 0.00 | 0.82 | 0.00 | 0.01 | 0.82 |
| Volume+GCS | 0.92 | 0.36 | 40.77 | 0.06 | 1.00 | 0.09 | 0.22 | 0.68 |
| Volume+GLS | 0.48 | 0.20 | 41.90 | 0.11 | 1.00 | 0.18 | 0.53 | 0.70 |
| Volume+T1 Pre | 0.31 | 0.16 | 42.77 | 0.20 | 1.00 | 0.34 | 1.00 | 0.66 |
| Volume+T1 Post | 0.31 | 0.16 | 42.74 | 0.20 | 1.00 | 0.33 | 1.00 | 0.65 |
| Volume+ECV | 0.54 | 0.15 | 42.61 | 0.18 | 1.00 | 0.30 | 1.00 | 0.68 |
| Volume+T2 Avg | 0.13 | 0.34 | 41.33 | 0.08 | 1.00 | 0.13 | 0.33 | 0.70 |
| Volume+Mass/Vol | 0.44 | 0.54 | 38.93 | 0.02 | 1.00 | 0.03 | 0.07 | 0.71 |
| Mass+LVEF | 0.10 | 0.15 | 42.06 | 0.12 | 1.00 | 0.20 | 0.63 | 0.69 |
| Mass+RVEF | 0.02 | 0.07 | 30.53 | 0.00 | 0.03 | 0.00 | 0.00 | 0.88 |
| Mass+GCS | 0.07 | 0.27 | 40.39 | 0.04 | 1.00 | 0.07 | 0.17 | 0.72 |
| Mass+GLS | 0.09 | 0.19 | 41.83 | 0.11 | 1.00 | 0.17 | 0.50 | 0.71 |
| Mass+T1 Pre | 0.74 | 0.25 | 43.50 | 0.34 | 1.00 | 0.67 | 1.00 | 0.66 |
| Mass+T1 Post | 0.59 | 0.32 | 43.83 | 0.44 | 1.00 | 1.00 | 1.00 | 0.63 |
| Mass+ECV | 0.41 | 0.34 | 43.96 | 0.50 | 1.00 | 1.00 | 1.00 | 0.62 |
| Mass+T2 Avg | 0.84 | 0.16 | 40.10 | 0.04 | 1.00 | 0.06 | 0.14 | 0.66 |
| Mass+Mass/Vol | 0.07 | 0.57 | 38.98 | 0.02 | 1.00 | 0.03 | 0.07 | 0.71 |
| LVEF+RVEF | 0.06 | 0.29 | 33.96 | 0.00 | 0.27 | 0.00 | 0.00 | 0.85 |
| LVEF+GCS | 0.30 | 0.88 | 41.63 | 0.09 | 1.00 | 0.15 | 0.42 | 0.66 |
| LVEF+GLS | 0.48 | 0.86 | 43.65 | 0.38 | 1.00 | 0.86 | 1.00 | 0.65 |
| LVEF+T1 Pre | 0.62 | 0.50 | 44.41 | 0.93 | 1.00 | 1.00 | 1.00 | 0.59 |
| LVEF+T1 Post | 0.66 | 0.46 | 44.31 | 0.74 | 1.00 | 1.00 | 1.00 | 0.61 |
| LVEF+ECV | 0.29 | 0.32 | 43.88 | 0.46 | 1.00 | 1.00 | 1.00 | 0.63 |
| LVEF+T2 Avg | 0.39 | 0.61 | 42.03 | 0.12 | 1.00 | 0.20 | 0.61 | 0.64 |
| LVEF+Mass/Vol | 0.04 | 0.34 | 38.33 | 0.01 | 1.00 | 0.02 | 0.05 | 0.74 |
| RVEF+GCS | 0.01 | 0.02 | 34.25 | 0.00 | 0.34 | 0.00 | 0.00 | 0.82 |
| RVEF+GLS | 0.01 | 0.01 | 35.16 | 0.00 | 0.97 | 0.00 | 0.01 | 0.80 |
| RVEF+T1 Pre | 0.04 | 0.01 | 34.49 | 0.00 | 0.41 | 0.00 | 0.01 | 0.83 |
| RVEF+T1 Post | 0.05 | 0.01 | 35.14 | 0.00 | 0.88 | 0.00 | 0.01 | 0.80 |
| RVEF+ECV | 0.03 | 0.01 | 32.36 | 0.00 | 0.09 | 0.00 | 0.00 | 0.85 |
| RVEF+T2 Avg | 0.05 | 0.02 | 34.83 | 0.00 | 0.57 | 0.00 | 0.01 | 0.83 |
| RVEF+Mass/Vol | 0.01 | 0.02 | 30.49 | 0.00 | 0.03 | 0.00 | 0.00 | 0.88 |
| GCS+GLS | 0.06 | 0.16 | 41.42 | 0.08 | 1.00 | 0.13 | 0.35 | 0.65 |
| GCS+T1 Pre | 0.26 | 0.09 | 41.43 | 0.08 | 1.00 | 0.13 | 0.35 | 0.67 |
| GCS+T1 Post | 0.28 | 0.10 | 41.50 | 0.09 | 1.00 | 0.14 | 0.37 | 0.66 |
| GCS+ECV | 0.17 | 0.07 | 40.95 | 0.06 | 1.00 | 0.10 | 0.25 | 0.71 |
| GCS+T2 Avg | 0.88 | 0.19 | 40.43 | 0.05 | 1.00 | 0.07 | 0.17 | 0.70 |
| GCS+Mass/Vol | 0.02 | 0.22 | 37.61 | 0.01 | 1.00 | 0.01 | 0.03 | 0.75 |
| GLS+T1 Pre | 0.63 | 0.29 | 43.67 | 0.39 | 1.00 | 0.96 | 1.00 | 0.64 |
| GLS+T1 Post | 0.83 | 0.25 | 43.48 | 0.33 | 1.00 | 0.66 | 1.00 | 0.63 |
| GLS+ECV | 0.23 | 0.18 | 42.92 | 0.22 | 1.00 | 0.38 | 1.00 | 0.65 |
| GLS+T2 Avg | 0.53 | 0.98 | 42.29 | 0.14 | 1.00 | 0.24 | 0.98 | 0.64 |
| GLS+Mass/Vol | 0.02 | 0.18 | 37.36 | 0.01 | 1.00 | 0.01 | 0.03 | 0.79 |
| T1 Pre+T1 Post | 0.75 | 0.79 | 44.78 | 1.00 | 1.00 | 1.00 | 1.00 | 0.58 |
| T1 Pre+ECV | 0.88 | 0.64 | 44.68 | 1.00 | 1.00 | 1.00 | 1.00 | 0.60 |
| T1 Pre+T2 Avg | 0.63 | 0.80 | 42.22 | 0.14 | 1.00 | 0.23 | 0.80 | 0.66 |
| T1 Pre+Mass/Vol | 0.26 | 0.51 | 38.86 | 0.02 | 1.00 | 0.03 | 0.06 | 0.74 |
| T1 Post+ECV | 0.95 | 0.80 | 44.83 | 1.00 | 1.00 | 1.00 | 1.00 | 0.56 |
| T1 Post+T2 Avg | 0.31 | 0.72 | 42.16 | 0.13 | 1.00 | 0.22 | 0.72 | 0.67 |
| T1 Post+Mass/Vol | 0.10 | 0.74 | 39.19 | 0.02 | 1.00 | 0.03 | 0.08 | 0.76 |
| ECV+T2 Avg | 0.64 | 0.21 | 40.63 | 0.05 | 1.00 | 0.08 | 0.20 | 0.70 |
| ECV+Mass/Vol | 0.12 | 0.85 | 39.27 | 0.02 | 1.00 | 0.04 | 0.08 | 0.74 |
| T2 Avg+Mass/Vol | 0.33 | 0.18 | 37.29 | 0.01 | 1.00 | 0.01 | 0.03 | 0.77 |
|  | **4-week prediction of Dysfunction (12 week)** | | | | | | | |
| Volume+Mass | 0.31 | 0.10 | 33.44 | 1.00 | 1.00 | 1.00 | 1.00 | 0.71 |
| Volume+LVEF | 0.47 | 0.49 | 30.82 | 0.48 | 1.00 | 0.15 | 1.00 | 0.82 |
| Volume+RVEF | 0.37 | 0.62 | 28.15 | 0.07 | 0.61 | 0.03 | 0.36 | 0.85 |
| Volume+GCS | 0.67 | 0.24 | 32.80 | 1.00 | 1.00 | 0.80 | 1.00 | 0.77 |
| Volume+GLS | 0.72 | 0.21 | 31.09 | 0.63 | 1.00 | 0.18 | 1.00 | 0.85 |
| Volume+T1 Pre | 0.12 | 0.17 | 32.80 | 1.00 | 1.00 | 0.79 | 1.00 | 0.71 |
| Volume+T1 Post | 0.06 | 0.30 | 30.39 | 0.33 | 1.00 | 0.12 | 1.00 | 0.80 |
| Volume+ECV | 0.35 | 0.12 | 33.69 | 1.00 | 1.00 | 1.00 | 1.00 | 0.67 |
| Volume+T2 Avg | 0.08 | 0.48 | 28.48 | 0.09 | 1.00 | 0.04 | 0.47 | 0.86 |
| Volume+Mass/Vol | 0.67 | 0.24 | 33.43 | 1.00 | 1.00 | 1.00 | 1.00 | 0.71 |
| Mass+LVEF | 0.06 | 0.42 | 30.63 | 0.40 | 1.00 | 0.13 | 1.00 | 0.78 |
| Mass+RVEF | 0.07 | 0.62 | 28.14 | 0.07 | 0.61 | 0.03 | 0.35 | 0.81 |
| Mass+GCS | 0.38 | 0.87 | 34.26 | 1.00 | 1.00 | 1.00 | 1.00 | 0.71 |
| Mass+GLS | 0.22 | 0.62 | 32.62 | 1.00 | 1.00 | 0.62 | 1.00 | 0.73 |
| Mass+T1 Pre | 0.41 | 0.82 | 34.86 | 1.00 | 1.00 | 1.00 | 1.00 | 0.68 |
| Mass+T1 Post | 0.14 | 0.76 | 31.48 | 1.00 | 1.00 | 0.24 | 1.00 | 0.78 |
| Mass+ECV | 1.00 | 0.84 | 36.41 | 1.00 | 1.00 | 1.00 | 1.00 | 0.54 |
| Mass+T2 Avg | 0.24 | 0.65 | 28.80 | 0.11 | 1.00 | 0.04 | 0.64 | 0.85 |
| Mass+Mass/Vol | 0.49 | 0.24 | 33.43 | 1.00 | 1.00 | 1.00 | 1.00 | 0.71 |
| LVEF+RVEF | 0.02 | 0.24 | 26.89 | 0.04 | 0.22 | 0.01 | 0.15 | 0.89 |
| LVEF+GCS | 0.04 | 0.08 | 30.39 | 0.33 | 1.00 | 0.12 | 1.00 | 0.83 |
| LVEF+GLS | 0.05 | 0.14 | 30.43 | 0.34 | 1.00 | 0.12 | 1.00 | 0.79 |
| LVEF+T1 Pre | 0.32 | 0.08 | 31.18 | 0.70 | 1.00 | 0.19 | 1.00 | 0.79 |
| LVEF+T1 Post | 0.70 | 0.06 | 26.35 | 0.03 | 0.15 | 0.01 | 0.10 | 0.86 |
| LVEF+ECV | 0.08 | 0.03 | 30.71 | 0.43 | 1.00 | 0.14 | 1.00 | 0.81 |
| LVEF+T2 Avg | 0.98 | 0.41 | 28.30 | 0.08 | 0.75 | 0.03 | 0.40 | 0.82 |
| LVEF+Mass/Vol | 0.02 | 0.04 | 29.72 | 0.21 | 1.00 | 0.08 | 1.00 | 0.83 |
| RVEF+GCS | 0.01 | 0.03 | 28.13 | 0.07 | 0.60 | 0.03 | 0.35 | 0.86 |
| RVEF+GLS | 0.01 | 0.04 | 27.69 | 0.06 | 0.40 | 0.02 | 0.25 | 0.88 |
| RVEF+T1 Pre | 0.28 | 0.02 | 27.64 | 0.05 | 0.38 | 0.02 | 0.24 | 0.86 |
| RVEF+T1 Post | 0.65 | 0.03 | 24.47 | 0.01 | 0.05 | 0.00 | 0.03 | 0.91 |
| RVEF+ECV | 0.09 | 0.02 | 27.71 | 0.06 | 0.40 | 0.02 | 0.25 | 0.88 |
| RVEF+T2 Avg | 0.64 | 0.23 | 27.36 | 0.05 | 0.31 | 0.02 | 0.20 | 0.87 |
| RVEF+Mass/Vol | 0.01 | 0.02 | 27.75 | 0.06 | 0.42 | 0.02 | 0.26 | 0.86 |
| GCS+GLS | 0.04 | 0.41 | 32.18 | 1.00 | 1.00 | 0.41 | 1.00 | 0.77 |
| GCS+T1 Pre | 0.98 | 0.27 | 33.65 | 1.00 | 1.00 | 1.00 | 1.00 | 0.71 |
| GCS+T1 Post | 0.32 | 0.39 | 30.73 | 0.44 | 1.00 | 0.14 | 1.00 | 0.79 |
| GCS+ECV | 0.48 | 0.16 | 34.26 | 1.00 | 1.00 | 1.00 | 1.00 | 0.72 |
| GCS+T2 Avg | 0.32 | 0.45 | 28.39 | 0.09 | 0.91 | 0.03 | 0.43 | 0.83 |
| GCS+Mass/Vol | 0.08 | 0.27 | 33.66 | 1.00 | 1.00 | 1.00 | 1.00 | 0.76 |
| GLS+T1 Pre | 0.96 | 0.13 | 32.25 | 1.00 | 1.00 | 0.43 | 1.00 | 0.79 |
| GLS+T1 Post | 0.17 | 0.04 | 25.35 | 0.01 | 0.08 | 0.01 | 0.06 | 0.89 |
| GLS+ECV | 0.17 | 0.07 | 32.15 | 1.00 | 1.00 | 0.40 | 1.00 | 0.77 |
| GLS+T2 Avg | 0.31 | 0.88 | 28.98 | 0.13 | 1.00 | 0.05 | 0.88 | 0.82 |
| GLS+Mass/Vol | 0.03 | 0.06 | 30.48 | 0.36 | 1.00 | 0.12 | 1.00 | 0.81 |
| T1 Pre+T1 Post | 0.07 | 0.51 | 31.11 | 0.65 | 1.00 | 0.18 | 1.00 | 0.79 |
| T1 Pre+ECV | 0.62 | 0.23 | 34.89 | 1.00 | 1.00 | 1.00 | 1.00 | 0.69 |
| T1 Pre+T2 Avg | 0.11 | 0.35 | 28.07 | 0.07 | 0.56 | 0.03 | 0.33 | 0.86 |
| T1 Pre+Mass/Vol | 0.83 | 0.17 | 32.83 | 1.00 | 1.00 | 0.85 | 1.00 | 0.71 |
| T1 Post+ECV | 0.14 | 0.04 | 29.99 | 0.25 | 1.00 | 0.09 | 1.00 | 0.81 |
| T1 Post+T2 Avg | 0.02 | 0.05 | 22.08 | 0.00 | 0.01 | 0.00 | 0.01 | 0.93 |
| T1 Post+Mass/Vol | 0.42 | 0.08 | 31.18 | 0.71 | 1.00 | 0.19 | 1.00 | 0.79 |
| ECV+T2 Avg | 0.77 | 0.14 | 26.89 | 0.04 | 0.22 | 0.01 | 0.15 | 0.87 |
| ECV+Mass/Vol | 0.51 | 0.88 | 34.97 | 1.00 | 1.00 | 1.00 | 1.00 | 0.67 |
| T2 Avg+Mass/Vol | 0.47 | 0.04 | 28.20 | 0.08 | 0.65 | 0.03 | 0.37 | 0.82 |
|  | **8-week prediction of Dysfunction (12 week)** | | | | | | | |
| Volume+Mass | 0.94 | 0.23 | 32.97 | 1.00 | 1.00 | 1.00 | 1.00 | 0.74 |
| Volume+LVEF | 0.04 | 0.11 | 27.94 | 0.08 | 1.00 | 0.03 | 1.00 | 0.85 |
| Volume+RVEF | 0.06 | 0.20 | 22.46 | 0.00 | 0.15 | 0.00 | 0.10 | 0.91 |
| Volume+GCS | 0.63 | 0.46 | 35.92 | 1.00 | 1.00 | 1.00 | 1.00 | 0.57 |
| Volume+GLS | 0.20 | 0.38 | 32.02 | 1.00 | 1.00 | 0.36 | 1.00 | 0.75 |
| Volume+T1 Pre | 0.33 | 0.63 | 35.08 | 1.00 | 1.00 | 1.00 | 1.00 | 0.67 |
| Volume+T1 Post | 0.27 | 0.34 | 32.17 | 1.00 | 1.00 | 0.41 | 1.00 | 0.77 |
| Volume+ECV | 0.29 | 0.70 | 34.31 | 1.00 | 1.00 | 1.00 | 1.00 | 0.68 |
| Volume+T2 Avg | 0.03 | 0.33 | 24.03 | 0.01 | 0.49 | 0.00 | 0.30 | 0.92 |
| Volume+Mass/Vol | 0.37 | 0.96 | 32.78 | 1.00 | 1.00 | 0.80 | 1.00 | 0.74 |
| Mass+LVEF | 0.04 | 0.14 | 27.58 | 0.07 | 1.00 | 0.02 | 1.00 | 0.85 |
| Mass+RVEF | 0.08 | 0.15 | 20.83 | 0.00 | 0.06 | 0.00 | 0.04 | 0.91 |
| Mass+GCS | 0.17 | 0.18 | 34.28 | 1.00 | 1.00 | 1.00 | 1.00 | 0.70 |
| Mass+GLS | 0.08 | 0.21 | 30.78 | 0.69 | 1.00 | 0.15 | 1.00 | 0.76 |
| Mass+T1 Pre | 0.90 | 0.23 | 33.66 | 1.00 | 1.00 | 1.00 | 1.00 | 0.69 |
| Mass+T1 Post | 0.08 | 0.66 | 32.86 | 1.00 | 1.00 | 1.00 | 1.00 | 0.74 |
| Mass+ECV | 0.92 | 0.47 | 33.91 | 1.00 | 1.00 | 1.00 | 1.00 | 0.67 |
| Mass+T2 Avg | 0.08 | 0.36 | 24.13 | 0.01 | 0.54 | 0.00 | 0.33 | 0.93 |
| Mass+Mass/Vol | 0.09 | 0.90 | 32.77 | 1.00 | 1.00 | 0.78 | 1.00 | 0.74 |
| LVEF+RVEF | 0.02 | 0.73 | 24.39 | 0.01 | 0.73 | 0.00 | 0.40 | 0.92 |
| LVEF+GCS | 0.07 | 0.05 | 30.42 | 0.47 | 1.00 | 0.12 | 1.00 | 0.79 |
| LVEF+GLS | 0.06 | 0.18 | 30.90 | 0.84 | 1.00 | 0.16 | 1.00 | 0.78 |
| LVEF+T1 Pre | 0.41 | 0.07 | 30.72 | 0.64 | 1.00 | 0.14 | 1.00 | 0.80 |
| LVEF+T1 Post | 0.03 | 0.05 | 28.05 | 0.09 | 1.00 | 0.03 | 1.00 | 0.84 |
| LVEF+ECV | 0.30 | 0.08 | 30.88 | 0.81 | 1.00 | 0.16 | 1.00 | 0.79 |
| LVEF+T2 Avg | 0.26 | 0.44 | 24.48 | 0.01 | 0.86 | 0.00 | 0.43 | 0.93 |
| LVEF+Mass/Vol | 0.03 | 0.11 | 29.88 | 0.30 | 1.00 | 0.08 | 1.00 | 0.81 |
| RVEF+GCS | 0.03 | 0.02 | 20.22 | 0.00 | 0.04 | 0.00 | 0.03 | 0.95 |
| RVEF+GLS | 0.01 | 0.03 | 24.47 | 0.01 | 0.86 | 0.00 | 0.43 | 0.93 |
| RVEF+T1 Pre | 0.42 | 0.02 | 23.48 | 0.01 | 0.31 | 0.00 | 0.20 | 0.92 |
| RVEF+T1 Post | 0.06 | 0.03 | 23.68 | 0.01 | 0.36 | 0.00 | 0.23 | 0.88 |
| RVEF+ECV | 0.22 | 0.02 | 24.44 | 0.01 | 0.80 | 0.00 | 0.42 | 0.91 |
| RVEF+T2 Avg | 0.55 | 0.08 | 19.32 | 0.00 | 0.02 | 0.00 | 0.02 | 0.96 |
| RVEF+Mass/Vol | 0.03 | 0.02 | 23.93 | 0.01 | 0.45 | 0.00 | 0.28 | 0.91 |
| GCS+GLS | 0.21 | 0.55 | 32.50 | 1.00 | 1.00 | 0.56 | 1.00 | 0.73 |
| GCS+T1 Pre | 0.39 | 0.75 | 35.22 | 1.00 | 1.00 | 1.00 | 1.00 | 0.63 |
| GCS+T1 Post | 0.08 | 0.65 | 32.87 | 1.00 | 1.00 | 1.00 | 1.00 | 0.74 |
| GCS+ECV | 0.25 | 0.58 | 34.14 | 1.00 | 1.00 | 1.00 | 1.00 | 0.68 |
| GCS+T2 Avg | 0.02 | 0.34 | 24.07 | 0.01 | 0.51 | 0.00 | 0.31 | 0.95 |
| GCS+Mass/Vol | 0.10 | 0.47 | 32.27 | 1.00 | 1.00 | 0.45 | 1.00 | 0.72 |
| GLS+T1 Pre | 0.93 | 0.10 | 32.28 | 1.00 | 1.00 | 0.45 | 1.00 | 0.75 |
| GLS+T1 Post | 0.06 | 0.11 | 30.15 | 0.37 | 1.00 | 0.10 | 1.00 | 0.81 |
| GLS+ECV | 0.89 | 0.18 | 32.59 | 1.00 | 1.00 | 0.61 | 1.00 | 0.72 |
| GLS+T2 Avg | 0.08 | 0.79 | 25.03 | 0.02 | 1.00 | 0.01 | 0.79 | 0.93 |
| GLS+Mass/Vol | 0.06 | 0.30 | 31.66 | 1.00 | 1.00 | 0.28 | 1.00 | 0.75 |
| T1 Pre+T1 Post | 0.80 | 0.14 | 29.81 | 0.29 | 1.00 | 0.08 | 1.00 | 0.78 |
| T1 Pre+ECV | 0.28 | 0.61 | 34.19 | 1.00 | 1.00 | 1.00 | 1.00 | 0.68 |
| T1 Pre+T2 Avg | 0.05 | 0.45 | 24.48 | 0.01 | 0.87 | 0.00 | 0.43 | 0.94 |
| T1 Pre+Mass/Vol | 0.76 | 0.58 | 32.47 | 1.00 | 1.00 | 0.54 | 1.00 | 0.76 |
| T1 Post+ECV | 0.63 | 0.17 | 32.17 | 1.00 | 1.00 | 0.41 | 1.00 | 0.73 |
| T1 Post+T2 Avg | 0.17 | 0.54 | 24.69 | 0.01 | 1.00 | 0.00 | 0.52 | 0.92 |
| T1 Post+Mass/Vol | 0.09 | 0.25 | 31.31 | 1.00 | 1.00 | 0.21 | 1.00 | 0.85 |
| ECV+T2 Avg | 0.03 | 0.41 | 24.34 | 0.01 | 0.68 | 0.00 | 0.38 | 0.94 |
| ECV+Mass/Vol | 0.57 | 0.96 | 32.78 | 1.00 | 1.00 | 0.79 | 1.00 | 0.73 |
| T2 Avg+Mass/Vol | 0.15 | 0.05 | 24.91 | 0.01 | 1.00 | 0.00 | 0.66 | 0.92 |
| Chiseq: The two parametere model is compared to the indicated single parameter model. | | | | | | | | |
