# Supplementary figures and images for: Early detection of myocardial changes with and without dexrazoxane using serial magnetic resonance imaging in a pre-clinical mouse model
Source: Cardiooncology. 2021 Jun 16;7:23. doi: 10.1186/s40959-021-00109-8 (PMC8207719; doi:10.1186/s40959-021-00109-8)

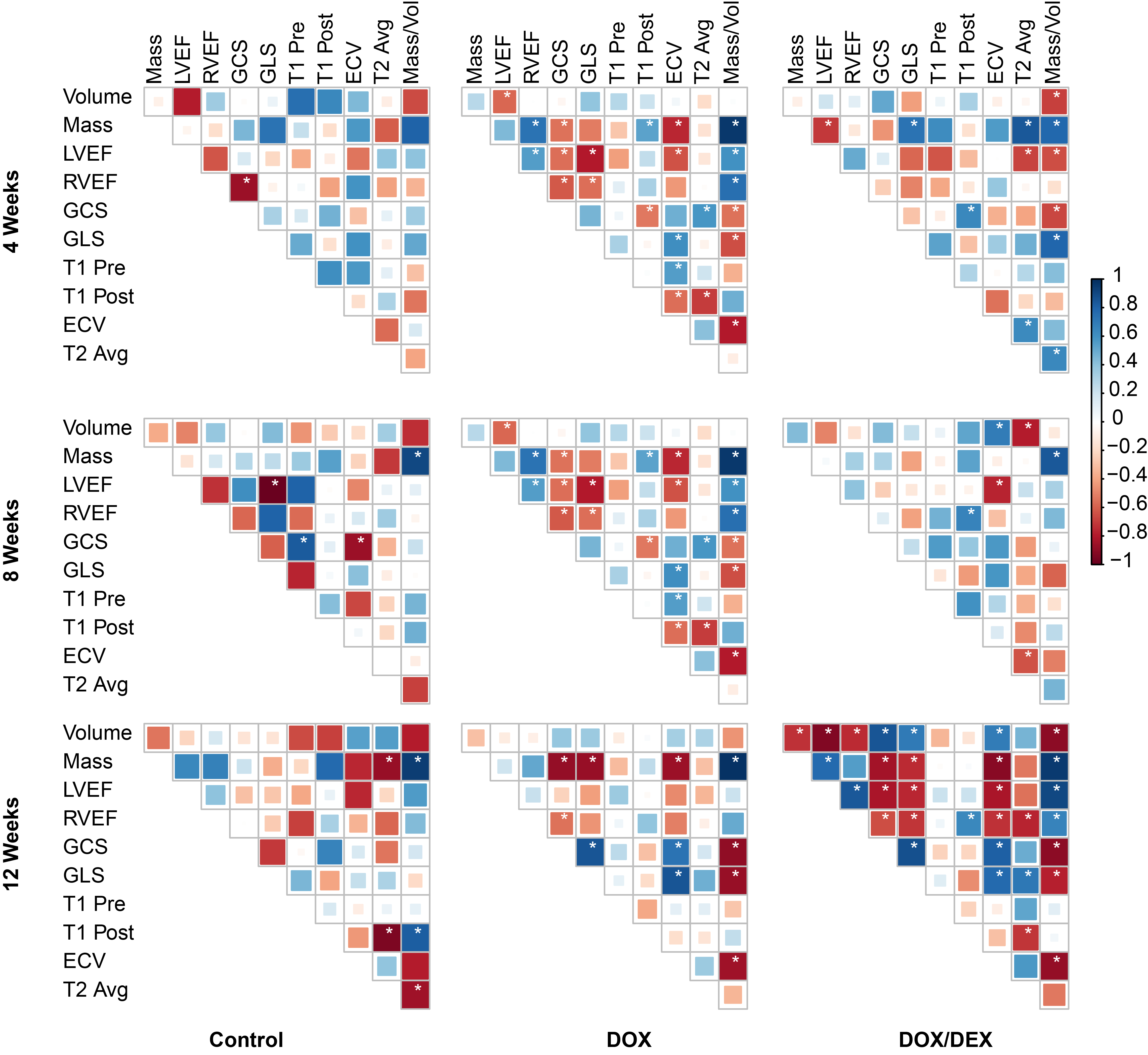

Supplement: Supplementary file 5 — Additional file 5. [file 40959_2021_109_MOESM5_ESM.png]

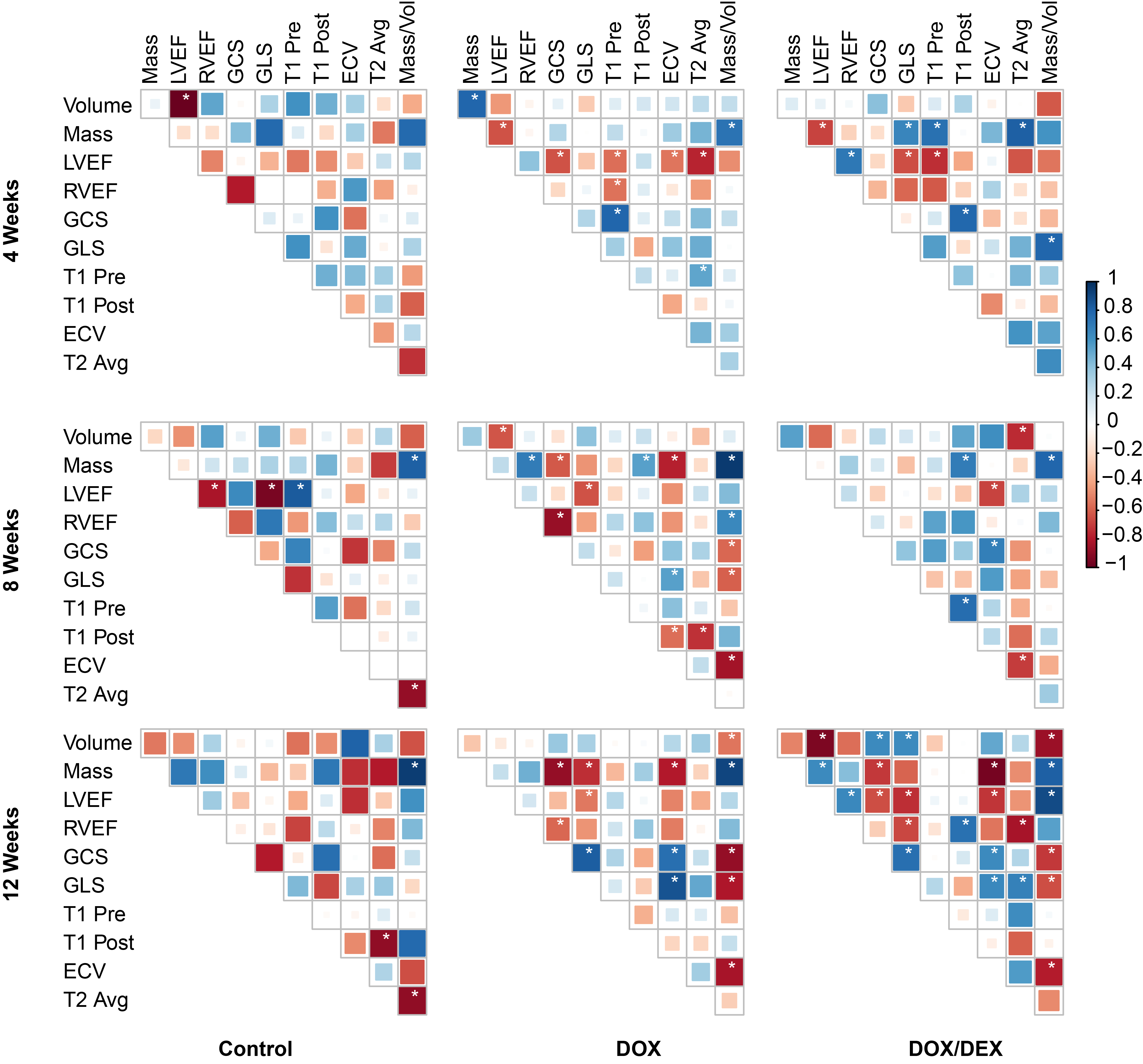

Supplement: Supplementary file 6 — Additional file 6. [file 40959_2021_109_MOESM6_ESM.png]

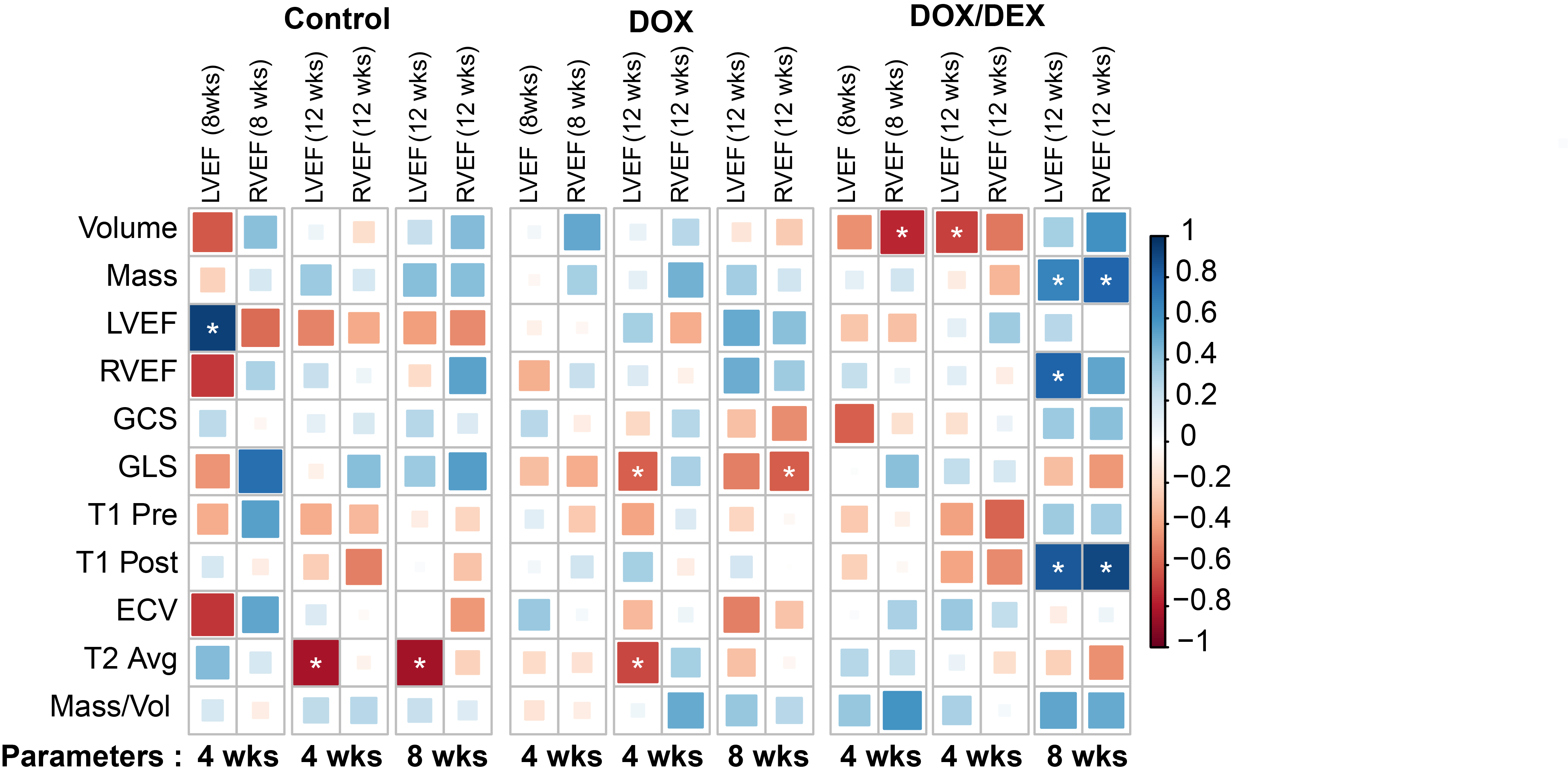

Supplement: Supplementary file 7 — Additional file 7. [file 40959_2021_109_MOESM7_ESM.png]

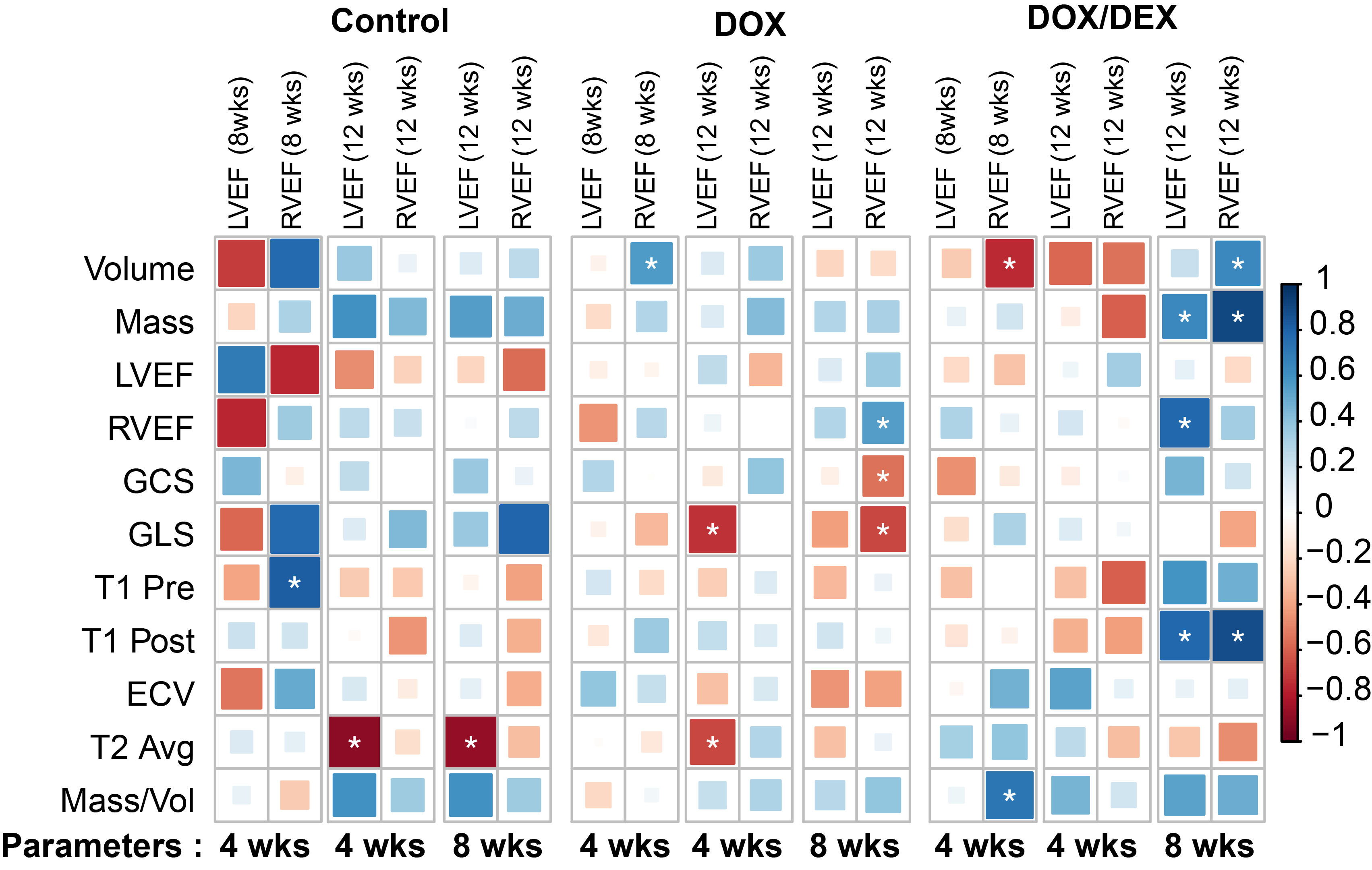

Supplement: Supplementary file 8 — Additional file 8. [file 40959_2021_109_MOESM8_ESM.png]
